# Impact of Harvest on Switchgrass Leaf Microbial Communities

**DOI:** 10.3390/genes13010022

**Published:** 2021-12-22

**Authors:** Esther Singer, Elizabeth M. Carpenter, Jason Bonnette, Tanja Woyke, Thomas E. Juenger

**Affiliations:** 1 Joint Genome Institute, Berkeley, CA 94720, USA; twoyke@lbl.gov; 2 Lawrence Berkeley National Laboratory, Berkeley, CA 94710, USA; EMCarpenter@lbl.gov; 3 Department of Integrative Biology, University of Texas Austin, Austin, TX 78712, USA; jebonnette@hotmail.com

**Keywords:** switchgrass, plant microbial community composition, harvest, leaf metabarcoding data, fungi, phyllosphere, leaves, plant genotypes

## Abstract

Switchgrass is a promising feedstock for biofuel production, with potential for leveraging its native microbial community to increase productivity and resilience to environmental stress. Here, we characterized the bacterial, archaeal and fungal diversity of the leaf microbial community associated with four switchgrass (*Panicum virgatum*) genotypes, subjected to two harvest treatments (annual harvest and unharvested control), and two fertilization levels (fertilized and unfertilized control), based on 16S rRNA gene and internal transcribed spacer (ITS) region amplicon sequencing. Leaf surface and leaf endosphere bacterial communities were significantly different with Alphaproteobacteria enriched in the leaf surface and Gammaproteobacteria and Bacilli enriched in the leaf endosphere. Harvest treatment significantly shifted presence/absence and abundances of bacterial and fungal leaf surface community members: Gammaproteobacteria were significantly enriched in harvested and Alphaproteobacteria were significantly enriched in unharvested leaf surface communities. These shifts were most prominent in the upland genotype DAC where the leaf surface showed the highest enrichment of Gammaproteobacteria, including taxa with 100% identity to those previously shown to have phytopathogenic function. Fertilization did not have any significant impact on bacterial or fungal communities. We also identified bacterial and fungal taxa present in both the leaf surface and leaf endosphere across all genotypes and treatments. These core taxa were dominated by *Methylobacterium*, *Enterobacteriaceae*, and *Curtobacterium*, in addition to *Aureobasidium*, *Cladosporium*, *Alternaria* and *Dothideales*. Local core leaf bacterial and fungal taxa represent promising targets for plant microbe engineering and manipulation across various genotypes and harvest treatments. Our study showcases, for the first time, the significant impact that harvest treatment can have on bacterial and fungal taxa inhabiting switchgrass leaves and the need to include this factor in future plant microbial community studies.

## 1. Introduction

Plant microbial communities vary in structure and function across diverse plant species [1], genotypes [2], ecosystems [3], soil types [4], and soil and plant treatments [5], as showcased by many amplicon sequencing studies. Significant effort has been made in the study of plant microbial communities below ground, illustrating impacts of rhizosphere and root endosphere microbial communities on plant health [6,7] and crop yield [8,9,10,11], with less data available on plant-associated microbial communities above ground. Although leaves generally host lower microbial biomass than roots, some of the insights obtained from phyllosphere microbial community studies show that leaf microbiota provide plant growth-promoting benefits. For example, a strain of *Enterobacter* sp. was shown to increase biomass production in *Populus* spp. [12] and various *Methylobacter* spp. produce ACC (1-aminocyclopropane-1-carboxylate deaminase) deaminases that degrade the precursor of the phytohormone ethylene [13]. Microorganisms of plant leaves also contribute to global biogeochemical cycles. For instance, various *Methylobacter* spp. can oxidize and assimilate methanol released by plant leaves as a byproduct of pectin formation during cell wall synthesis [14], and leaf-colonizing Cyanobacteria and Gammaproteobacteria spp. aid in N_2_ fixation in the plant species *Carludovica drudei, Grias cauliflora* and *Costus leavis* [15]. Consistent occurrences of core leaf taxa over time, as were observed in switchgrass and miscanthus, suggest the potential for microbial community manipulation and management [16]. Most studies have investigated the bacterial/archaeal fraction of the plant microbial community, whereas plant-associated fungal data are less abundant, and studies that include both bacteria and fungi are even more scarce [17]. Hence, there is a need to advance insights into bacterial, archaeal and fungal phyllosphere communities in order to understand and make targeted use of the many beneficial or antagonistic interactions between plants and their holistic microbial communities.

Similar to many other plant microbial communities, the switchgrass microbial community has been shown to influence plant function, and its composition differs to varying degrees by environmental characteristics [18,19,20,21], plant compartment (shoots vs. roots) [2,16,22,23], climate and seasonality [16,24,25], and switchgrass genotype [2,26]. As a biofuel crop, switchgrass plantings are commonly treated, for example, with soil amendments; however, few studies have investigated the effect of such management practices on the switchgrass microbial community, especially on the phyllosphere microbial community. Grady et al., for example, found that N fertilization of the soil did not show any impact on bacterial and archaeal community structure in the phyllosphere [16], and Bowsher et al. demonstrated that leaf nitrogen did indeed impact fungal community composition (although that observation may have been partially caused by an edge effect resulting from the experimental layout) [27]. The potential impact of N fertilization on bacterial, archaeal or fungal phyllosphere microbial communities hence appears inconclusive and additional data is required.

Harvest practice, which represents another management practice commonly applied annually or biannually to perennial switchgrass, has not been studied as a potential driver of switchgrass microbial community composition [17]. Harvest dates vary by geographical location and associated switchgrass genotype, and are typically set upon senescence and the beginning of the winter season. Although the perennial crop efficiently recycles nutrients, multiple harvest events can cause stress to the plant, for example, due to the significant decrease in cellulose and lignin content of above-ground biomass [28]. It remains unclear whether the phyllosphere microbial community is qualitatively or quantitatively impacted by the timing or frequency of harvest events.

In this study, we ask two questions about the bacterial, archaeal and fungal communities inhabiting the leaf surface and leaf endosphere of switchgrass: (1) Are there differences in total and core microbial community composition between plants that are harvested annually vs. plants that are not harvested? (2) Are bacterial, archaeal and fungal community structures on different genotypes differently affected by harvest or fertilizer treatment? Improving our understanding of the phyllosphere microbial community will aid in arriving at sustainable management strategies that improve biomass quality, in addition to plant and ecosystem health.

## 2. Materials and Methods

### 2.1. Switchgrass Plants and Study Location

Switchgrass plants selected for this study include clonal divisions of 4 genotypes: Alamo-AP13, WBC, Summer-VS16 and DAC (Table S1). AP13 and WBC are representative southern lowland ecotypes, whereas Summer-VS16 and DAC are northern upland ecotypes. Rhizome divisions of these genotypes were obtained in the fall of 2011 and (with their original microbial community) transferred to a greenhouse located at the Brackenridge Field Lab facility of the University of Texas in Austin, TX (30°17′8.7″ N, 97°46′44.93″ W). Rhizomes were planted in 5-gallon pots with ProMix BX Mycorrhizae potting mix (Premier Tech Horticulture, Quakertown, PA, USA) and allowed to grow over winter in the greenhouse (14 h days). The resulting plants were then clonally propagated repeatedly during 2012 in an outdoor nursery in 1-gallon pots containing composted pine bark mulch (Leaf Landscape Supply, Austin, TX, USA) augmented with Osmocote 14-14-14 slow release fertilizer (The Scotts Company, Marysville, OH, USA). This is an inorganic fertilizer composed of 14% of nitrogen, phosphorus and potassium.

Ramets of each of the four genotypes were planted in a random block design in April 2013 outdoors in concrete cylinders (2 ft diameter by 4 ft height) containing Ranch Rose potting soil (Geo Growers, Austin, TX, USA) at the Pickle Research Center facility of the University of Texas in Austin, TX (PKL) (30°23′11.8″ N, 97°43′36.8″ W) (Figure S1, Figure S2). Plants were amply irrigated and allowed to establish during the 2013 growing season. Above-ground biomass was removed for half of the plants after plant senescence in the early winter of 2013 by cutting tillers approximately 10 cm above the soil surface with a reciprocating hedge trimmer. The other half of the plants were not harvested, i.e., their above-ground biomass naturally senesced and remained as standing material through the overwintering and into the establishment of green up and the generation of new tillers in the following year. In 2014, half of the plants were fertilized with urea at a rate equivalent to 70 lbs N/acre based on soil surface area of the cylinders. Fertilizer was applied on 2 May 2014, one month after all genotypes had emerged from winter dormancy, and again on 15 June 2014, just prior to panicle emergence of the lowland genotypes and just after flowering of the upland ecotypes. Leaf material for this study was collected in November 2014, before the harvested plants underwent their 2014 harvest event. Each genotype, harvest, and fertilizer treatment combination was represented by 6 replicates in this study, totaling 96 plants (4 genotypes × 2 harvest treatment levels × 2 fertilizer treatments × 6 biological replicates = 96) (Table S1). Leaf material from each of these 96 plants represent one data point each, where DNA extraction and sequencing were successful.

### 2.2. Leaf Sampling and Library Preparation

Leaf samples from all experimental plants representing four different genotypes were collected in November 2014. Flag leaves were harvested and washed with a buffer (0.1X PBS buffer, 0.1% Triton X-100) to obtain leaf surface (LS) samples. In the field, 2 g of leaf material was washed with 45 mL of PBS buffer on a tabletop shaker for 15 min at 200 rpm. Leaf wash solution with epiphyte communities was frozen at −20 °C with 10% glycerol to prevent cell burst during storage. Leaf washes were then filtered onto 0.2 µm GTTP filter membranes (Whatman, Maidstone, UK). For leaf endosphere (LE) samples, leaves were washed with tap water, sterilized with 3% sodium hypochlorite solution, rinsed with sterile MilliQ water, ground with liquid nitrogen and frozen at −80 °C until DNA extraction. Triplicate control samples to showcase the success of the sterilization process were generated by reiterating the leaf washing step after the leaves were sterilized. Amplification of DNA and subsequent sequencing failed. Control samples were hence not included in the data analysis of this study.

### 2.3. DNA Extraction, Amplification and Sequencing

DNA extraction was performed using the MoBio Power Water kit (MoBio, Carlsbad, CA, USA). For leaf washes we used ½ of the filter membranes for DNA extraction. DNA concentrations were quantified using a Pico Green assay (Thermo Fisher, Waltham, MA, USA). Samples were prepared for sequencing the V4 region of the 16S rRNA gene (V4 iTags) using 515F and 816R primers, and the ITS2 region (ITS) using ITS9 and ITS4 primers, using standard JGI protocols (http://1ofdmq2n8tc36m6i46scovo2e.wpengine.netdna-cdn.com/wp-content/uploads/2017/08/iTag-Sample-Preparation-for-Illumina-Sequencing-SOP-v1.0.pdf, accessed on 21 December 2021). iTag sequencing was performed according to JGI’s standard procedures: iTag V4 and ITS amplicons were diluted to 10 nM, quantified by quantitative PCR and sequenced on the Illumina MiSeq platform (reagent kit v.3; Illumina Inc., San Diego, CA, USA) as described in [2].

### 2.4. α- and β-Diversity Analyses

α-diversity analysis (observed ASVs) was performed in QIIME2 [29,30]. Principal coordinate analysis to show grouping of samples by plant compartment was computed in R [31] based on Bray–Curtis distances and using the *vegdist* function of the Vegan package. Factor contribution to % community variance was assessed using the *adonis* function with 999 permutations as part of the Vegan package in R [32]. β-diversity analysis based on weighted UniFrac and the Kruskal–Wallis test was performed in QIIME2. Tree construction for UniFrac calculations was achieved by aligning ASV sequences with MAFFT v. 7.221 [33] and calculating branch lengths using FastTree 2 [34]. ASV abundance in grouped samples is based on average relative abundance in the respective samples. Differentially abundant ASVs with ≥100 reads across groups were obtained by applying the Analysis of Composition of Microbiomes (ANCOM) algorithm in QIIME2. Permutational ANOVAs (PERMANOVAs) were performed with the function ‘adonis’ in the Vegan package as described in [2]. Because the categorical variable ‘genotype’ is nested within ‘ecotype’, we used the ‘strata’ argument within the ‘adonis’ function (Table S2). Local core ASVs were determined as bacterial/fungal ASVs shared between 50% and 100% of samples using QIIME2. Related 16S rRNA gene sequences were identified in the NCBI database using the blastn algorithm. Matching sequences with 100% sequence identity over 100% of the gene length were then used in added literature research in order to retrieve information about the corresponding organism’s potential lifestyle.

## 3. Results and Discussion

We used amplicon sequencing to analyze the bacterial, archaeal and fungal communities associated with leaf surface and leaf endosphere compartments of four switchgrass genotypes that were either harvested or not harvested, and in the presence and absence of fertilization. Plant genotypes showed phenotypic differences at the time of sample collection (Figures S1 and S4), which can be linked to different growth season durations and earlier onset of senescence in the upland genotypes. Lowland ecotypes acclimated to warmer, wetter southern climates had ~10-fold larger biomass compared to the upland ecotypes (Figure S4) when grown in Austin, which is a commonly observed phenological difference between the ecotypes [35].

Leaf endosphere samples from unharvested plants yielded few sequences for both bacteria and fungi. Although leaf endosphere and surface samples were obtained using separate protocols, we did not separate samples by treatment during sample processing, DNA extraction or amplicon library creation. Because we did not measure microbial biomass, we can only speculate about whether unharvested LE samples failed during sequencing due to a biological (lower bacteria/fungal biomass in leaves of unharvested plants) or a technical cause (unknown batch effect). Thus, the harvest treatment was analyzed only in the context of leaf surface (LS) samples.

### 3.1. Sequencing Summary

In total, we sequenced 97 leaf endosphere (LE) and 96 leaf surface (LS) samples at the end of the growing season in 2014. The number of sequences per sample after applying the dada2 amplicon sequence variant (ASV) pipeline [36] ranged from 1670 to 69,027 sequences per sample. After removing sequences that were attributed to chloroplasts and mitochondria, or that had unassigned taxonomic classification, we filtered ASVs that were not present in ≥5 samples with ≥10 reads. α rarefaction curves are shown in Figure S3. Because leaf endosphere samples from unharvested plants yielded few sequences for both bacteria and fungi, we decided to exclude these samples from the analysis (Table S1).

### 3.2. Microbial Community Assembly Was Impacted by Leaf Compartment, Harvest Treatment and Genotype

In order to gain insight into microbial leaf community structure in switchgrass and factors for microbial community assembly, we analyzed the distribution of bacterial/archaeal and fungal ASVs correlated to factors considered in the experimental design, i.e., genotype, ecotype, harvest treatment, fertilization level and their interactions. We found that microbial community variance was explained by various biotic and abiotic factors and factor interactions (Table S2, Figure S5). When taking all samples into consideration, plant compartment contributed the most to overall bacterial/archaeal community variance (~27%), followed by genotype (nested within ecotype) (~2%). This is consistent with findings from other studies that reported distinct bacterial/archaeal community composition between different tissue types of switchgrass plants [2,16,22,23]. Fertilizer did not affect plant above-ground biomass, leaf microbial community or root/soil microbial community as determined in the complementary switchgrass root microbial community study [2], likely because the planting soil provided the plants with ample nutrients and/or because N fertilization may have been too moderate to observe an impact.

**Figure 1:**
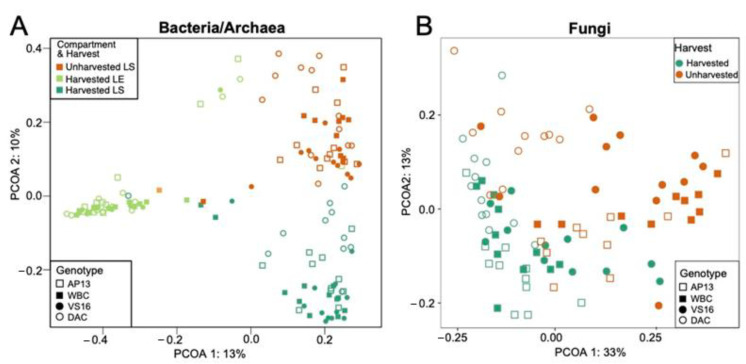
Principal coordinate analysis of (**A**) bacterial/archaeal leaf surface (LS) and leaf endosphere (LE) and (**B**) fungal leaf surface communities at the ASV level. Colors denote compartments and harvest treatments, shapes indicate plant genotype.

Within the LS communities, genotype and harvest treatment combined contributed the most variance in both bacterial/archaeal (~17%) and fungal (~7%) communities (Figure 1, Table S2, Figure S5). Fertilization did not impact bacterial or fungal communities in LS samples. This finding is consistent with a study from Grady et al., who also did not see a significant impact of fertilization on switchgrass and miscanthus leaf surface microbial community, and only a small, although significant, influence on soil bacteria/archaeal communities [16]. Although Bowsher et al. did find a small, but significant, correlation between N fertilization and foliar leaf surface community variance, it is not clear to what degree this result was caused by their experimental design rather than by the N treatment [27].

In the leaf endosphere (LE) samples, we found that ecotype was a significant driver of bacteria/archaeal community variance (~20%) and that ecotype and genotype significantly impacted the fungal community (~5% and ~7%, respectively) (Table S2). We also interrogated the fertilization impact among the harvested LE samples and did not find a significant impact of fertilization on bacteria/archaeal or fungal communities. Because many of the unharvested LE samples failed for the bacterial/archaeal and fungal communities, as mentioned above, we did not investigate any potential correlations between harvest and communities.

Microbial communities have rarely been separately analyzed and contrasted between LS and LE, and no study to date has done so for switchgrass plants. Hence, comparisons to other studies for the above-mentioned analyses were hampered. Because plant compartment, genotype and ecotype, and harvest treatment displayed distinct clustering of microbial community diversity and composition, we continued our analyses with samples grouped according to these factors.

**Figure 2:**
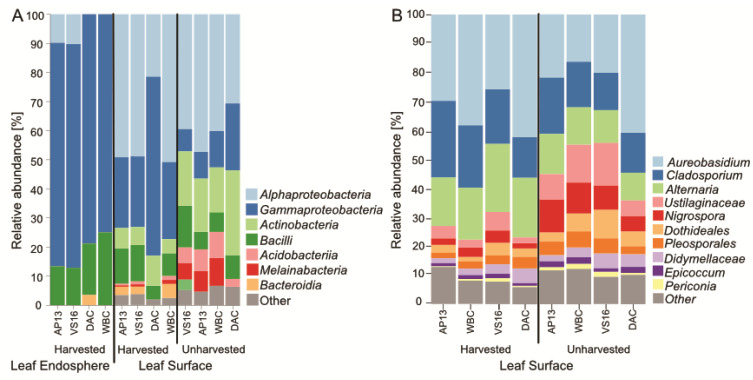
Bacterial/archaeal community composition in leaf endosphere and leaf surface (**A**) and fungal community composition in the leaf surface (**B**). Harvested and non-harvested samples, and genotypes, are contrasted. Coloring is by taxonomic order.

We detected 45 bacterial and three archaeal classes belonging to 19 bacterial and two archaeal phyla across all samples (Figure 2A). Similar to other studies, archaea did not represent a dominant fraction of reads and displayed little diversity [17]. Both leaf surface and leaf endosphere were dominated by Proteobacteria, Bacilli, and, to a lesser extent, Actinobacteria. Alphaproteobacteria, Gammaproteobacteria, Actinobacteria and Bacteroidia are dominant taxonomic groups in most plant microbial communities e.g., [2,37,38,39]. The switchgrass leaf microbial community was dominated by many of the same phyla that have been detected in other plant microbial community studies [16,27,37] and Alphaproteobacteria and Gammaproteobacteria have been observed to dominate switchgrass leaf microbial communities throughout the seasons [16]. Interestingly, we observed relatively more Actinobacteria, Acidobacteriia and Melainabacteria in unharvested LS samples across all genotypes (Figure 2A). The fungal communities were dominated by Ascomycota and, to lesser extent, contained *Basidiomycota*, which is an observation common for plant-associated fungal communities, as seen, for example, in a switchgrass study conducted over several seasons [27]. We detected 129 fungal genera (Figure 2B) belonging to four classes (Figure S6). Fungal communities were dominated by genera of the Dothideomycetes class, including *Aureobasidium, Cladosporium* and *Alternaria*, which are ubiquitous plant colonizing genera [40,41,42].

**Figure 3:**
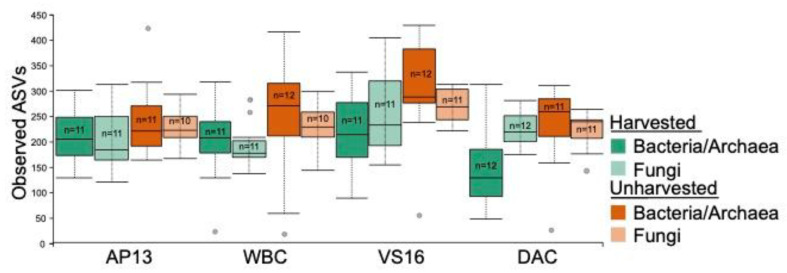
Bacterial/archaeal and fungal α-diversity in the leaf surface depicted as the number of observed ASVs and grouped by harvest treatment and genotype. α-diversity was significantly lower in harvested vs. unharvested samples (bacteria: *p* = 0.0006; fungi: *p* = 0.000002). It was significantly lower in DAC than in the other genotypes (*p* = 0.002–0.03) for bacteria, and significantly higher in VS16 than in the other genotypes (*p* = 0.009) for fungi.

The number of observed bacterial/archaeal ASVs was significantly lower in harvested vs. unharvested leaf surface samples (Bacteria/Archaea: *p* = 8.9 × 10^−4^; Fungi: *p* = 0.004) (Figure 3). Genotype did not significantly impact the number of observed bacterial/archaeal ASVs; however, it did impact fungal communities (*p =* 0.006). Observed ASVs did not significantly vary for different fertilizer treatments or ecotypes in either bacterial/archaeal or fungal communities.

### 3.3. Differentially Abundant ASVs in Leaves from Harvested Plants Were Related to Pathogens

**Figure 4:**
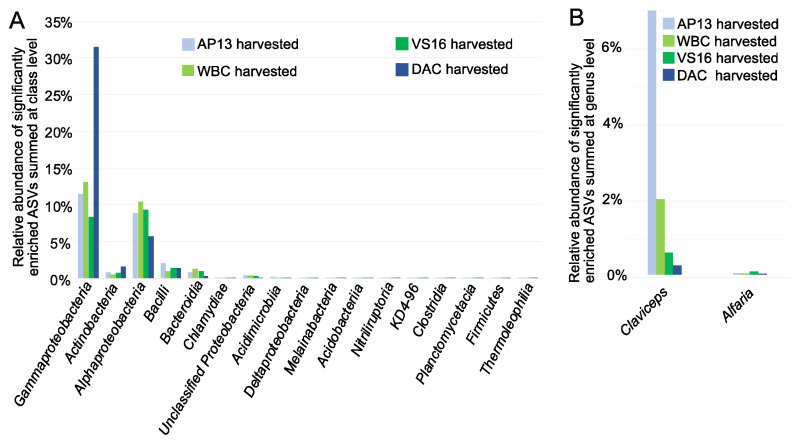
Analysis of composition (ANCOM) of bacterial (**A**) and fungal ASVs (**B**) associated with leaves of harvested vs. unharvested switchgrass. Listed are taxa that showed significantly higher abundances across all four genotypes by harvest level with >100 reads. Relative abundances are averages across samples from respective genotypes.

We asked whether bacterial/archaeal or fungal taxa were significantly enriched between leaf compartments, harvest levels and ecotypes/genotypes. Because leaf compartment contributed the most to the bacterial community differences (Table S2), we compared leaf surface and leaf endosphere bacterial/archaeal communities and found that 85 bacterial ASVs were significantly more abundant in the leaf surface compared to the leaf endosphere (Table S3A). Less than 10 ASVs were more abundant in the endosphere than the leaf surface, and were represented by <100 reads. Microbial taxa significantly enriched by harvest level included Gammaproteobacteria and Bacteroidia, which were both enriched in harvested plants, whereas Actinobacteria, Acidobacteriia and Melainabacteria were enriched in unharvested plants (Figure 2). The greatest enrichment was of Gammaproteobacteria, which was 10–30% more abundant in harvested plants of all genotypes (Figures S7 and S8). AP13 was the least enriched with Gammaproteobacteria, whereas DAC was the most enriched (Figure 4A, Table S3B). Harvest was also correlated with significant differential enrichment of Alphaproteobacteria, Actinobacteria and Bacilli ASVs (Figure 4, Figure S7).

The abundances of two fungal ASVs, belonging to *Claviceps* and *Alfaria* genera, differed between harvested and unharvested leaf surface samples. Although the *Claviceps* ASV was significantly enriched in harvested AP13 leaf surface samples, the *Alfaria* ASV was most abundant in the harvested VS16 leaf surface (Figure 4B). Harvested leaf surface samples showed significantly enriched fungal taxa *Claviceps* and *Alfaria* compared to unharvested samples. The *Claviceps* ASV was 100% identical to ASVs commonly found in grass disease studies in the southern United States [43]. The *Alfaria* ASV was 100% identical to ASVs belonging to the *Stachybotriaceae* family that have been associated with plant pathogenicity [44].

LS samples from unharvested plants displayed a number of significantly enriched taxa that belonged to Alphaproteobacteria, mostly *Methylobacterium, Bradyrhizobium* and genera within the *Xanthobacteriaceae* family, and totaled nearly 8% of the total community. Alignment of our sequences to those in the literature resulted in 100% identity over 100% sequence length with organisms that were shown to fix nitrogen (e.g., MT534083.1), to be microsymbionts (e.g., MT468658.1) or to display other plant growth-promoting potential (e.g., MT360236.1). Leaf surface samples from harvested plants were enriched in *Gammaproteobacteria*, specifically *Enterobacteriaceae, Xanthomonadaceae* and *Pseudomonas* taxa, accounting for nearly 12% of the total community. Some of these sequences matched those from organisms found to be phytopathogens and ubiquitous colonizers with 100% identity. For example, we found four *Xanthomonadaceae* ASVs in our harvested leaf surface samples that were 100% identical to *Xanthomonas axonopodis* (MK818495.1), which was found to cause bacterial complex diseases (BCD) [45]. Additionally, we found two ASVs with 100% identity over 100% query coverage to *Pantoea dispersa* (HQ683985.1), an endophyte isolated from various dicot plants. In contrast, in the unharvested leaf surface samples, we found six *Xanthomonadaceae* ASVs that shared 100% identity over 100% coverage with *Bradyrhizobium* sp. Iri (AB933528.1), *Bradyrhizobium* sp. strain Lcos102 (MT468658.1), *Bradyrhizobium japonicum* strain 15 (KU298505.1) and *Bradyrhizobium neotropicale* strain APP82 (MT534079.1), which are all root-nodule forming bacteria and hence associated with N_2_ fixation and plant growth promotion [46]. Interestingly, harvested switchgrass displayed reduced microbial diversity and we found significantly enriched taxa that shared 100% sequence identity with *Xanthomonadaceae* strains that demonstrated phytopathogenic behavior [45].

Genotypes less adapted to the planting environment displayed earlier senescence and a higher abundance of ASVs related to taxa shown to cause plant disease in other studies. Speculatively, this observation of increased ASVs that may fulfill pathogenic functions on the leaves may be the result of a compromised immune system in plants that had experienced harvest or tissue damage caused by harvesting, which is a route for infection, or perhaps because unharvested plants retained a microbial community that is resistant to colonization by pathogens. Time series sampling and plant gene expression studies (such as [27]) planned around harvest events would provide insights into plant stress level and respective bacterial and fungal community responses associated with harvesting.

### 3.4. The Local Switchgrass Leaf Surface Bacterial Core Was Smaller than the Fungal Core

The prevalence of ASVs shared between 50% and 100% of samples (i.e., the local core microbial community) was computed to identify ASVs that were present in our switchgrass leaf study regardless of genotype and plant treatment (Table S4). We refer to our core ASVs as “local core ASVs” because our study design was limited to one location and time point. In the LE, only two ASVs were shared among 50% of samples and they were both classified as *Pseudomonas*. These two ASVs accounted for >20% relative abundance of the LE microbial community (Figure S9). We examined the local core microbial community of LE samples by switchgrass ecotype because ecotype significantly impacted the LE microbial community (Table S2). The local LE core bacteria included only a few ASVs with high relative abundances: the upland core consisted of one *Pseudomonas* ASV, the lowland core consisted of three *Pseudomonas* ASVs. At 65% of samples, only one ASV was found in each ecotype core, with the upland core represented by a different *Pseudomonas* ASV than the lowland core (Table S5).

**Figure 5:**
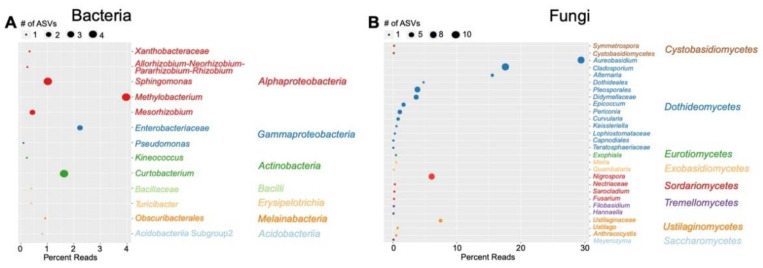
Leaf surface core microbial community in (**A**) bacteria shared among 80% of samples and (**B**) fungi shared among 90% of samples. ASVs are clustered by genus, coloring is by class. Percent reads of individual genera constitute the relative abundance in the LS compartment.

The local LS core bacterial microbial community across 90% of samples was composed of ubiquitous leaf genera, including four *Methylobacterium* ASVs, two *Enterobacteriaceae* ASVs, four *Curtobacterium* ASVs and four *Sphingomonas* ASVs (Table S6A). The local core bacterial microbial community was slightly larger in unharvested plants compared to harvested plants, whereas the taxonomic composition of the local core bacterial microbial community was not statistically significantly different between unharvested and harvested plants (Table S6A, Figure S10). The local fungal leaf surface core microbial community was comprised of eight classes, including 38 *Dothideomycetes* ASVs, 10 *Sordariomycetes* ASVs, four *Ustilaginomycetes* ASVs, two *Tremellomycetes* ASVs, two *Exobasidiomycetes* ASVs, one *Cystobasidiomycetes* ASV, one *Eurotiomycetes* ASV and one *Saccharomycetes* ASV (Figure 5).

The local LS fungal core microbial community displayed larger diversity at the genus level and included a much larger fraction of the total leaf surface community (Figure 5, Table S6B). In 50% of LS samples, 7.1% of the bacterial community and 92.1% of the fungal community was shared (Table S4B). In 95% of samples, ~76.9% of fungal ASVs were shared (Table S7B). Fungal ASVs shared among 90% of LS samples were dominated by genera of the Ascomycota: *Aureobasidium, Alternaria* and *Cladosporium* together accounted for ~62% of the total LS community (Figure 5, Table S6B). These genera have previously been identified as local core genera in switchgrass roots [2]. *Aureobasidium, Cladosporium* and *Nigrospora* displayed the most ASV diversity (7–8 ASVs per genus) (Figure 5). Of 59 local core fungal ASVs, 50 were Ascomycota and the remaining nine ASVs were Basidiomycota (Figure 5). Ascomycota have previously been found to dominate fungal switchgrass leaf communities [27,47,48]. Most local core genera belonged to the Dothideomycetes class; the remainder of the local core ASVs were relatively evenly distributed among *Cystobasidiomycetes, Eurotiomycetes, Exobasidiomycetes, Sordariomycetes, Saccharomycetes, Tremellomyceetes* and *Ustilaginomycetes*, with each class containing 1–4 ASVs (Figure 5). Bowsher et al. [27] also found a relatively high abundance of Dothideomycetes sequences in the late growing season and various studies have found this genus to be a globally important leaf taxon as it comprises the largest taxonomic, ecological and functional diversity of fungi and includes endophytes, mutualists and pathogens to agricultural crops [49,50,51,52,53,54]. Various local fungal LS core genera detected in our study have previously been detected on and inside switchgrass leaves. For example, *Alternaria* and *Epicoccum* spp. were isolated from the leaf endosphere of switchgrass plants, and in a subsequent inoculation study, were shown to impact biomass production [48]. Differences in local core fungal communities between harvested and unharvested plants were not significant (Table S6B, Figure S11).

The differences in local bacterial and fungal LS core microbial communities are striking and suggest that bacterial LS colonization may be driven more by host-specific leaf characteristics that could be heterogeneously impacted by environmental factors; this would render the LS habitat dynamic and challenging for bacteria, thereby limiting the bacterial biomass [37]. Furthermore, fungal colonization pathways may be shared among a large fraction of the fungal community, fungal attachment to leaves may be stronger, and fungal resistance to daily changes more robust, hence leading to a relatively large core on the LS. Similar bacterial and fungal core community trends have been observed in estuarine seagrass leaf microbial communities. Although different estuaries showed distinct core bacterial communities, the core fungal communities were largely shared, following a precipitation event and across a salinity gradient [55] or across plant compartments, seasons and sites [42]. The fact that local fungal core taxa are largely observed across different switchgrass genotypes provides an opportunity to identify plant growth-promoting fungal candidates that can be applied to various switchgrass variants, which is a research priority for sustainable agriculture.

## 4. Summary and Conclusions

Plant root microbial communities from a large diversity of host plants have been well studied and have transformed plant and soil management practices. With the recognition of the vast microbial diversity in soils and their functional importance came the realization that conventional agricultural techniques are often harmful to the balance of the native soil and root microbial communities. For example, pesticides were found to negatively impact bacterial diversity and nitrification rates [56] and synthetic fertilizer is known to suppress long-lasting relationships between plants and nitrogen-fixing bacteria [57]. Furthermore, native root-associated bacteria were shown to save a plant from sudden-wilt disease [58], and soil microbes have been found to display varying abilities to confer drought tolerance [59]. Because soil and root microbial communities represent a wealth of genetic and functional diversity with applications far beyond agriculture, much focus has also been on the development of new methods and instrumentation targeted towards accelerating our understanding of plant root microbial communities [60].

Compared to the large body of literature surrounding soil and plant root microbial communities, there is relatively little published leaf microbial community data, regardless of plant species or environment, although leaves are easy to sample comprehensively. Our findings in this study highlight that the practice of agronomic harvesting of grasses can significantly impact both bacterial and fungal switchgrass LS microbial community composition, and, to a lesser non-significant degree, result in changes to (local) core community composition. The striking impact of this treatment on the total LS bacterial and fungal communities may provide insight into the stress level and broader plant–microbial community–soil ecosystem health, in addition to plant phenotypic features that contribute to the establishment of leaf microbial communities. It may be that harvested plants recruit more microorganisms from the soil or from their crown, whereas unharvested plants obtain additional microorganisms from past years’ vegetation, through which they grow as new leaf material establishes each spring. The number of harvest events (once or twice per year) was previously shown to impact nutrient cycling within the plant and in the soil [61]; however, long-term impact on the plant microbial community may play an additional important role in plant health. Our findings may give rise to additional future studies in that direction. We also observed distinct impacts of harvest on bacterial/archaeal and fungal community composition based on the switchgrass genotype. Although all genotypes showed increases in gammaproteobacterial ASVs in harvested plants, genotypes less adapted to the planting environment displayed earlier senescence and a higher abundance of ASVs related to taxa shown to cause plant disease in other studies. Finally, we showed that bacterial and fungal community trends can behave very differently depending on the investigated experimental factor.

Our study presents data from one site and one time point; however, switchgrass genotypes in this study also represent differences in developmental phase, with upland ecotypes transitioning to a fall senescence earlier than lowland ecotypes at our southern field location. Hence our study also suggests temporal leaf surface community dynamics that are confounded with harvest treatment and host genetics. Because temporal [16] and geographical [62] factors tend to strongly impact plant (core) microbial community composition in both agricultural and non-agricultural settings, the impact of harvest treatment compared to spatiotemporal factors remains to be disclosed. Although amplicon studies generate interesting hypotheses about plant microbial community relationships, complementary plant gene expression and functional metagenomics and metatranscriptomic datasets will allow us to gain insights into microbial activity as a function of host genotype and gene expression, and environmental changes, which will also be necessary for improving ecological models in the face of climate change.

## Data Availability

All relevant data files can be found at https://genome.jgi.doe.gov/portal/switchgrassmicrobiome/switchgrassmicrobiome.home.html, accessed on 21 December 2021.

## Supplementary Materials

The following are available online at https://www.mdpi.com/article/10.3390/genes13010022/s1: Figure S1: Above-ground phenotypic differences between switchgrass plants from harvested and unharvested lowland (AP13, WBC) and upland (VS16, DAC) plants. (A) Harvested AP13, (B) unharvested AP13, (C) harvested WBC, (D) unharvested WBC, (E) harvested VS16, (F) unharvested VS16, (G) harvested DAC, (H) unharvested DAC. Figure S2: Experimental setup. 96 plants were individually placed in concrete cylinders preventing exchange between plants. Figure S3: Rarefaction curves based on (A) 16S rRNA gene sequencing and on (B) ITS sequencing. Figure S4: Biomass differences between switchgrass genotypes/ecotypes. Figure S5: Relative contribution of experimental factors to microbial community variance. (B) = Bacteria; (F) = Fungi. Figure S6: Community composition of fungi in phyllosphere samples at the class level grouped by harvest level and genotype. Figure S7: Taxa enriched in harvested vs. unharvested switchgrass phyllosphere samples by genotype. (A) AP13 genotype, lowland ecotype; (B) WBC genotype, lowland ecotype; (C) VS16 genotype, upland ecotype; (D) DAC genotype, upland ecotype. Figure S8: Log fold change in significantly enriched bacterial ASVs by harvest level in the leaf surface. Figure S9: Bacterial core microbial community composition shared among 50% of all, leaf surface (LS), and leaf endosphere (LE) samples. Figure S10: Core microbial community in the LS by harvest level. Figure S11: Relative abundance of fungal taxa in harvested vs. unharvested leaf surface samples. Table S1: Numbers of 16S rRNA gene and ITS amplicon sequencing samples that passed sequencing QC steps. Table S2: Permuted multivariate analysis of variance (PERMANOVA) tables for all hypothesis tests for difference in community β-diversity. Only significant results are shown. “Genotype (Ecotype)” denotes that the analysis was performed as Genotype nested within Ecotype. Table S3: Number of significantly enriched ASVs in the phyllosphere compared to leaf endosphere grouped by genus (A) and average frequency of significantly different abundant ASVs per genotype across phyllosphere samples (B). Taxa were classified at the highest level possible. Table S4: Core bacterial microbial community across a range of sample fractions. Computation was conducted across all samples (A), phyllosphere (B) and leaf endosphere (C). Core community composition is depicted in Figure 5. Table S5: Core bacterial ASVs in 65% of upland or lowland leaf endosphere samples. No ASVs were shared in >65% of lowland samples and no ASVs were shared in >75% of upland samples. Table S6: Core bacterial (A) and fungal (B) ASVs in 90% of samples by harvest level. Table S7: Core fungal ASVs across a range of sample fractions. Computation is conducted across all samples (A) and the leaf surface (B). The leaf endosphere was omitted from this analysis since many LE samples failed. Core community composition is depicted in Figure 5.

## References

[1] Berendsen R.L., Pieterse C.M.J., Bakker P.A.H.M. (2012). The Rhizosphere Microbiome and Plant Health. Trends Plant Sci..

[2] Singer E., Bonnette J., Kenaley S.C., Woyke T., Juenger T.E. (2019). Plant Compartment and Genetic Variation Drive Microbiome Composition in Switchgrass Roots. Environ. Microbiol. Rep..

[3] Ramirez K.S., Snoek L.B., Koorem K., Geisen S., Bloem L.J., ten Hooven F., Kostenko O., Krigas N., Manrubia M., Caković D. (2019). Range-Expansion Effects on the Belowground Plant Microbiome. Nat. Ecol. Evol..

[4] Schreiter S., Ding G.-C., Heuer H., Neumann G., Sandmann M., Grosch R., Kropf S., Smalla K. (2014). Effect of the Soil Type on the Microbiome in the Rhizosphere of Field-Grown Lettuce. Front. Microbiol..

[5] Pii Y., Borruso L., Brusetti L., Crecchio C., Cesco S., Mimmo T. (2016). The Interaction between Iron Nutrition, Plant Species and Soil Type Shapes the Rhizosphere Microbiome. Plant Physiol. Biochem..

[6] Wallenstein M.D. (2017). Managing and Manipulating the Rhizosphere Microbiome for Plant Health: A Systems Approach. Rhizosphere.

[7] Bano S.A., Uzair B. (2021). Arbuscular Mycorrhizal Fungi (AMF) for Improved Plant Health and Production. Microbial Biotechnology in Crop Protection.

[8] Kumar A., Dubey A. (2020). Rhizosphere Microbiome: Engineering Bacterial Competitiveness for Enhancing Crop Production. J. Adv. Res..

[9] Tall S., Meyling N.V. (2018). Probiotics for Plants? Growth Promotion by the Entomopathogenic Fungus Beauveria Bassiana Depends on Nutrient Availability. Microb. Ecol..

[10] Nieto-Jacobo M.F., Steyaert J.M., Salazar-Badillo F.B., Nguyen D.V., Rostás M., Braithwaite M., De Souza J.T., Jimenez-Bremont J.F., Ohkura M., Stewart A. (2017). Environmental Growth Conditions of Trichoderma Spp. Affects Indole Acetic Acid Derivatives, Volatile Organic Compounds, and Plant Growth Promotion. Front. Plant Sci..

[11] Senthil Kumar C.M., Jacob T.K., Devasahayam S., Thomas S., Geethu C. (2018). Multifarious Plant Growth Promotion by an Entomopathogenic Fungus Lecanicillium Psalliotae. Microbiol. Res..

[12] Taghavi S., Garafola C., Monchy S., Newman L., Hoffman A., Weyens N., Barac T., Vangronsveld J., van der Lelie D. (2009). Genome Survey and Characterization of Endophytic Bacteria Exhibiting a Beneficial Effect on Growth and Development of Poplar Trees. Appl. Environ. Microbiol..

[13] Fedorov D.N., Ekimova G.A., Doronina N.V., Trotsenko Y.A. (2013). 1-Aminocyclopropane-1-Carboxylate (ACC) Deaminases from Methylobacterium Radiotolerans and Methylobacterium Nodulans with Higher Specificity for ACC. FEMS Microbiol. Lett..

[14] Zhang C., Wang M.-Y., Khan N., Tan L.-L., Yang S. (2021). Potentials, Utilization, and Bioengineering of Plant Growth-Promoting Methylobacterium for Sustainable Agriculture. Sustainability.

[15] Fürnkranz M., Wanek W., Richter A., Abell G., Rasche F., Sessitsch A. (2008). Nitrogen Fixation by Phyllosphere Bacteria Associated with Higher Plants and Their Colonizing Epiphytes of a Tropical Lowland Rainforest of Costa Rica. ISME J..

[16] Grady K.L., Sorensen J.W., Stopnisek N., Guittar J., Shade A. (2019). Assembly and Seasonality of Core Phyllosphere Microbiota on Perennial Biofuel Crops. Nat. Commun..

[17] Hestrin R., Lee M.R., Whitaker B.K., Pett-Ridge J. (2021). The Switchgrass Microbiome: A Review of Structure, Function, and Taxonomic Distribution. Phytobiomes J..

[18] Soman C., Keymer D.P., Kent A.D. (2018). Edaphic Correlates of Feedstock-associated Diazotroph Communities. GCB Bioenergy.

[19] Whitaker B.K., Reynolds H.L., Clay K. (2018). Foliar Fungal Endophyte Communities Are Structured by Environment but Not Host Ecotype in *Panicum Virgatum* (Switchgrass). Ecology.

[20] Brodsky O.L., Shek K.L., Dinwiddie D., Bruner S.G., Gill A.S., Hoch J.M., Palmer M.I., McGuire K.L. (2019). Microbial Communities in Bioswale Soils and Their Relationships to Soil Properties, Plant Species, and Plant Physiology. Front. Microbiol..

[21] Giauque H., Connor E.W., Hawkes C.V. (2019). Endophyte Traits Relevant to Stress Tolerance, Resource Use and Habitat of Origin Predict Effects on Host Plants. New Phytol..

[22] Bahulikar R.A., Torres-Jerez I., Worley E., Craven K., Udvardi M.K. (2014). Diversity of Nitrogen-Fixing Bacteria Associated with Switchgrass in the Native Tallgrass Prairie of Northern Oklahoma. Appl. Environ. Microbiol..

[23] Roley S.S., Xue C., Hamilton S.K., Tiedje J.M., Robertson G.P. (2019). Isotopic Evidence for Episodic Nitrogen Fixation in Switchgrass (*Panicum virgatum* L.). Soil Biol. Biochem..

[24] Chen H., Yang Z.K., Yip D., Morris R.H., Lebreux S.J., Cregger M.A., Klingeman D.M., Hui D., Hettich R.L., Wilhelm S.W. (2019). One-Time Nitrogen Fertilization Shifts Switchgrass Soil Microbiomes within a Context of Larger Spatial and Temporal Variation. PLoS ONE.

[25] Sawyer A., Staley C., Lamb J., Sheaffer C., Kaiser T., Gutknecht J., Sadowsky M.J., Rosen C. (2019). Cultivar and Phosphorus Effects on Switchgrass Yield and Rhizosphere Microbial Diversity. Appl. Microbiol. Biotechnol..

[26] Singer E., Bonnette J., Woyke T., Juenger T.E. (2019). Conservation of Endophyte Bacterial Community Structure Across Two Panicum Grass Species. Front. Microbiol..

[27] Bowsher A.W., Benucci G.M.N., Bonito G., Shade A. (2021). Seasonal Dynamics of Core Fungi in the Switchgrass Phyllosphere, and Co-Occurrence with Leaf Bacteria. Phytobiomes J..

[28] Mohammed Y.A., Raun W., Kakani G., Zhang H., Taylor R., Desta K.G., Jared C., Mullock J., Bushong J., Sutradhar A. (2015). Nutrient Sources and Harvesting Frequency on Quality Biomass Production of Switchgrass (*Panicum virgatum* L.) for Biofuel. Biomass Bioenergy.

[29] Bolyen E., Rideout J.R., Dillon M.R., Bokulich N.A., Abnet C.C., Al-Ghalith G.A., Alexander H., Alm E.J., Arumugam M., Asnicar F. (2019). Reproducible, Interactive, Scalable and Extensible Microbiome Data Science Using QIIME 2. Nat. Biotechnol..

[30] Estaki M., Jiang L., Bokulich N.A., McDonald D., González A., Kosciolek T., Martino C., Zhu Q., Birmingham A., Vázquez-Baeza Y. (2020). QIIME 2 Enables Comprehensive End-to-End Analysis of Diverse Microbiome Data and Comparative Studies with Publicly Available Data. Curr. Protoc. Bioinform..

[31] R Foundation for Statistical Computing (2019). R: A Language and Environment for Statistical Computing.

[32] Oksanen J., Blanchet F.G., Friendly M., Kindt R., Legendre P., McGlinn D., Minchin P.R., O’Hara R.B., Simpson G.L., Solymos P. (2020). Vegan: Community Ecology Package.

[33] Katoh K., Standley D.M. (2013). MAFFT Multiple Sequence Alignment Software Version 7: Improvements in Performance and Usability. Mol. Biol. Evol..

[34] Price M.N., Dehal P.S., Arkin A.P. (2010). FastTree 2—Approximately Maximum-Likelihood Trees for Large Alignments. PLoS ONE.

[35] Porter C.L. (1966). An Analysis of Variation Between Upland and Lowland Switchgrass, *Panicum virgatum* L., in Central Oklahoma. Ecology.

[36] Callahan B.J., McMurdie P.J., Rosen M.J., Han A.W., Johnson A.J.A., Holmes S.P. (2016). DADA2: High-Resolution Sample Inference from Illumina Amplicon Data. Nat. Methods.

[37] Vorholt J.A. (2012). Microbial Life in the Phyllosphere. Nat. Rev. Microbiol..

[38] Coleman-Derr D., Desgarennes D., Fonseca-Garcia C., Gross S., Clingenpeel S., Woyke T., North G., Visel A., Partida-Martinez L.P., Tringe S.G. (2016). Plant Compartment and Biogeography Affect Microbiome Composition in Cultivated and Native *Agave* Species. New Phytol..

[39] Bulgarelli D., Garrido-Oter R., Münch P.C., Weiman A., Dröge J., Pan Y., McHardy A.C., Schulze-Lefert P. (2015). Structure and Function of the Bacterial Root Microbiota in Wild and Domesticated Barley. Cell Host Microbe.

[40] Leopold D.R., Busby P.E. (2020). Host Genotype and Colonist Arrival Order Jointly Govern Plant Microbiome Composition and Function. Curr. Biol..

[41] Arrigoni E., Antonielli L., Pindo M., Pertot I., Perazzolli M. (2018). Tissue Age and Plant Genotype Affect the Microbiota of Apple and Pear Bark. Microbiol. Res..

[42] Knapp D.G., Lázár A., Molnár A., Vajna B., Karácsony Z., Váczy K.Z., Kovács G.M. (2021). Above-ground Parts of White Grapevine Vitis Vinifera Cv. Furmint Share Core Members of the Fungal Microbiome. Environ. Microbiol. Rep..

[43] Alderman S.C., Halse R.R., White J.F. (2004). A Reevaluation of the Host Range and Geographical Distribution of Clavicepser Species in the United States. Plant Dis..

[44] Lombard L., Houbraken J., Decock C., Samson R.A., Meijer M., Réblová M., Groenewald J.Z., Crous P.W. (2016). Generic Hyper-Diversity in Stachybotriaceae. Pers. Int. Mycol. J..

[45] Choi O., Kang B., Lee Y., Kim S., Kwon J.-H., Lee J., Kim J. (2021). Bacterial Disease Complex Including Bleached Spot, Soft Rot, and Blight on Onion Seedlings Caused by Complex Infections. Plant Dis..

[46] Mortuza M.F., Tomooka N., Habibi S., Akatsu T., Djedidi S., Naito K., Ohkama-Ohtsu N., Yokoyama T. (2020). Multiphase Characterization of Wild Vigna Associated Root Nodule Bacteria from Japanese Subtropical Islands Unveiled Novel High Temperature Resistant Bradyrhizobium Strains Having High Symbiotic Compatibility with Soybean and Mungbean. Soil Sci. Plant Nutr..

[47] Lee M.R., Hawkes C.V. (2021). Plant and Soil Drivers of Whole-Plant Microbiomes: Variation in Switchgrass Fungi from Coastal to Mountain Sites. Phytobiomes J..

[48] Kleczewski N.M., Bauer J.T., Bever J.D., Clay K., Reynolds H.L. (2012). A Survey of Endophytic Fungi of Switchgrass (*Panicum virgatum*) in the Midwest, and Their Putative Roles in Plant Growth. Fungal Ecol..

[49] Yao H., Sun X., He C., Maitra P., Li X.-C., Guo L.-D. (2019). Phyllosphere Epiphytic and Endophytic Fungal Community and Network Structures Differ in a Tropical Mangrove Ecosystem. Microbiome.

[50] Qian X., Duan T., Sun X., Zheng Y., Wang Y., Hu M., Yao H., Ji N., Lv P., Chen L. (2018). Host Genotype Strongly Influences Phyllosphere Fungal Communities Associated with *Mussaenda pubescens* Var. *alba* (Rubiaceae). Fungal Ecol..

[51] Abdelfattah A., Li Destri Nicosia M.G., Cacciola S.O., Droby S., Schena L. (2015). Metabarcoding Analysis of Fungal Diversity in the Phyllosphere and Carposphere of Olive (*Olea europaea*). PLoS ONE.

[52] Izuno A., Kanzaki M., Artchawakom T., Wachrinrat C., Isagi Y. (2016). Vertical Structure of Phyllosphere Fungal Communities in a Tropical Forest in Thailand Uncovered by High-Throughput Sequencing. PLoS ONE.

[53] Schoch C.L., Crous P.W., Groenewald J.Z., Boehm E.W.A., Burgess T.I., de Gruyter J., de Hoog G.S., Dixon L.J., Grube M., Gueidan C. (2009). A Class-Wide Phylogenetic Assessment of Dothideomycetes. Stud. Mycol..

[54] Prior R., Feige A., Begerow D. (2017). Antagonistic Activity of the Phyllosphere Fungal Community. Sydowia Int. J. Mycol..

[55] Trevathan-Tackett S., Allnutt T., Sherman C., Richardson M., Crowley T., Macreadie P. (2020). Spatial Variation of Bacterial and Fungal Communities of Estuarine Seagrass Leaf Microbiomes. Aquat. Microb. Ecol..

[56] Jacobsen C.S., Hjelmsø M.H. (2014). Agricultural Soils, Pesticides and Microbial Diversity. Curr. Opin. Biotechnol..

[57] Tripathi S., Srivastava P., Devi R.S., Bhadouria R. (2020). Influence of Synthetic Fertilizers and Pesticides on Soil Health and Soil Microbiology. Agrochemicals Detection, Treatment and Remediation.

[58] Santhanam R., Luu V.T., Weinhold A., Goldberg J., Oh Y., Baldwin I.T. (2015). Native Root-Associated Bacteria Rescue a Plant from a Sudden-Wilt Disease That Emerged during Continuous Cropping. Proc. Natl. Acad. Sci. USA.

[59] Zolla G., Badri D.V., Bakker M.G., Manter D.K., Vivanco J.M. (2013). Soil Microbiomes Vary in Their Ability to Confer Drought Tolerance to Arabidopsis. Appl. Soil Ecol..

[60] Singer E., Vogel J.P., Northen T., Mungall C.J., Juenger T.E. (2021). Novel and Emerging Capabilities That Can Provide a Holistic Understanding of the Plant Root Microbiome. Phytobiomes J..

[61] Guretzky J.A., Biermacher J.T., Cook B.J., Kering M.K., Mosali J. (2011). Switchgrass for Forage and Bioenergy: Harvest and Nitrogen Rate Effects on Biomass Yields and Nutrient Composition. Plant Soil.

[62] Yeoh Y.K., Dennis P.G., Paungfoo-Lonhienne C., Weber L., Brackin R., Ragan M.A., Schmidt S., Hugenholtz P. (2017). Evolutionary Conservation of a Core Root Microbiome across Plant Phyla along a Tropical Soil Chronosequence. Nat. Commun..

