# Supplementary material for: Impact of Harvest on Switchgrass Leaf Microbial Communities
Source: Genes (Basel). 2021 Dec 22;13(1):22. doi: 10.3390/genes13010022 (PMC8774549; doi:10.3390/genes13010022)
Supplement: Supplementary file 1 [file genes-13-00022-s001.zip › genes-1450973-supplementary.pdf]

Supplementary Information

Supplementary Table S1 | Numbers of 16S rRNA gene and ITS amplicon sequencing samples that passed sequencing QC steps.

**Phyllosphere**	**AP13**		**WBC**		**VS16**		**DAC**	
	Bacteria/Archaea	Fungi	Bacteria/Archaea	Fungi	Bacteria/Archaea	Fungi	Bacteria/Archaea	Fungi
**Harvested + Fertilized**	5	5	6	6	5	5	6	6
**Harvested + Unfertilized**	6	6	5	5	6	6	6	6
**Unharvested + Fertilized**	6	5	6	5	6	5	6	6
**Unharvested + Unfertilized**	5	5	6	5	6	6	5	6
**Leaf endosphere**	**AP13**		**WBC**		**VS16**		**DAC**	
	Bacteria/Archaea	Fungi	Bacteria/Archaea	Fungi	Bacteria/Archaea	Fungi	Bacteria/Archaea	Fungi
**Harvested + Fertilized**	6	1	6	1	6	2	6	4
**Harvested + Unfertilized**	6	2	5	1	6	2	6	3
**Unharvested + Fertilized**	1	4	0	0	1	4	0	6
**Unharvested + Unfertilized**	1	3	1	1	0	4	0	5

Supplementary Table S2 | Permuted multivariate analysis of variance (PERMANOVA) tables for all hypothesis tests for difference in community beta diversity. Only significant results are shown. "Genotype (Ecotype)" denotes that the analysis was performed as Genotype nested within Ecotype.

**Bacteria**	**Factor**	**R2**	**p-value**
All	Compartment	27.3	0.001
	Genotype	1.63	0.007
Leaf surface	Harvest	7.4	0.001
	Genotype (Ecotype)	10.0	0.001
	Ecotype	5.3	0.001
Leaf endosphere	Ecotype	19.8	0.001
**Fungi**	**Factor**	**R2**	**p-value**
Leaf surface	Harvest	4.5	0.001
	Genotype	2.1	0.001
	Ecotype	1.8	0.007
Leaf endosphere	Ecotype	4.7	0.002
	Genotype	7.2	0.002

Supplementary Table S3 | Number of significantly enriched ASVs in the phyllosphere compared to leaf endosphere grouped by genus (A) and average frequency of significantly different abundant ASVs per genotype across phyllosphere samples (B). Taxa were classified at the highest level possible.

A)

**# of ASVs**	**Taxonomy**	**Phylum/Class**
2	*Acidobacteriia* Subgroup 2	*Acidobacteria*
2	*Bifidobacterium*	*Actinobacteria*
1	*Corynebacteriaceae*	*Actinobacteria*
2	*Kineococcus*	*Actinobacteria*
2	*Microbacteriaceae*	*Actinobacteria*
4	*Curtobacterium*	*Actinobacteria*
2	*Streptomyces*	*Actinobacteria*
2	*Chitinophagaceae*	*Bacteroidetes*

2	*Obscuribacterales*	*Cyanobacteria*
4	*Bacilli*	*Firmicutes*
2	*Thermobacillus xylanilyticus*	*Firmicutes*
5	*Lactobacillus*	*Firmicutes*
2	*Desulfosporosinus*	*Firmicutes*
2	*Turicibacter*	*Firmicutes*
5	*Methylobacterium*	*Alphaproteobacteria*
6	*Allorhizobium-Neorhizobium-Pararhizobium-Rhizobium*	*Alphaproteobacteria*
2	*Mesorhizobium*	*Alphaproteobacteria*
2	*Ochrobactrum*	*Alphaproteobacteria*
4	*Xanthobacteraceae*	*Alphaproteobacteria*
2	*Bradyrhizobium*	*Alphaproteobacteria*
5	*Sphingomonas*	*Alphaproteobacteria*
2	Uncultured *Alkalilimnicola* sp.	*Gammaproteobacteria*
6	*Massilia*	*Gammaproteobacteria*
4	*Ralstonia*	*Gammaproteobacteria*
2	*Enterobacteriaceae*	*Gammaproteobacteria*
4	*Pseudomonas*	*Gammaproteobacteria*
1	*Rhodanobacteraceae*	*Gammaproteobacteria*
6	*Xanthomonadaceae*	*Gammaproteobacteria*

B)

**Test-Statistic**	P_{FDR}	**AP13**	**WBC**	**VS16**	**DAC**	**Taxonomy**	**Phylum/Class**
25.4	0.01	984	752	721	**5151**	*Enterobacteriaceae*	*Gammaproteobacteria*
25.2	0.01	964	754	792	**5890**	*Enterobacteriaceae*	*Gammaproteobacteria*
23.7	0.02	**134**	40	11	27	*Methylopilaceae*	*Alphaproteobacteria*
23.5	0.02	**262**	**308**	47	36	*Xanthomonadaceae*	*Gammaproteobacteria*
22.8	0.02	**302**	**330**	62	39	*Xanthomonadaceae*	*Gammaproteobacteria*

22.2	0.02	145	43	**292**	**895**	*Allorhizobium-Neorhizobium-Pararhizobium-Rhizobium*	*Alphaproteobacteria*
22.1	0.02	0	0	0	**24**	*Acetobacteraceae*	*Alphaproteobacteria*
20.1	0.04	**108**	51	8	22	*Methylopilaceae*	*Alphaproteobacteria*

Supplementary Table S4 | Core bacteria across a range of sample fractions. Computation was conducted across all samples (A), phyllosphere (B) and leaf endosphere (C). Core community composition is depicted in Figure 5.

A)

Fraction of samples [%]	ASV count	Fraction of community [%]
50	44	2.1
55	20	1.0
60	13	0.6
70	1	0.05
75	1	0.05

80	0	0.0

B)

Fraction of samples [%]	ASV count	Fraction of community [%]
50	143	7.1
55	112	5.5
60	93	4.6
65	70	3.5
75	41	2.0
80	24	1.2
85	10	0.5
90	10	0.5
95	4	0.2
100	0	0.0

C)

Fraction of samples [%]	ASV count	Fraction of community [%]
50	2	21.1
55	2	21.1
60	1	13.5
65	1	13.5
70	0	0.0

Supplementary Table S5 | Core bacterial ASVs in 65% of upland or lowland leaf endosphere samples. No ASVs were shared in >65% of lowland samples and no ASVs were shared in >75% of upland samples.

**Core ASV in 65% of samples and associated ASV ID**	**# of ASVs in upland plants**	**# of ASVs in lowland plants**
Pseudomonas spp. (e9fd312214016f62f5e539b1291863a5)	1	0
Pseudomonas spp. (affa8b9097e979324b1a9dd9694e42cb)	0	1

Supplementary Table S6 | Core bacterial (A) and fungal (B) ASVs in 90% of samples by harvest level.
A

**Core bacterial genera/families in 90% of samples**	**# of ASVs in harvested plants**	**# of ASVs in unharvested plants**
Methylobacterium	4	5
Enterobacteriaceae	2	0
Curtobacterium	2	4
Sphingomonas	2	1
Pseudomonas	1	0
Mesorhizobium	1	0
Chitinophagaceae	1	0
Bradyrhizobium	0	2
Obscuribacterales	0	2
Acidobacteriia	0	2
**Total**	13	16

B

Core fungal genera/families in 90% of samples	# of ASVs in harvested plants	# of ASVs in unharvested plants
Cladosporium	8	8
Nigrospora	6	6
Aureobasidium	6	4
Ustilaginaceae	3	3
Pleosporales	5	5
Curvularia	2	2
Periconia	2	3
Meira	1	1
Filobasidium	1	1
Nectriaceae	1	0

Alternaria	1	1
Keissleriella	1	0
Epicoccum	1	0
Exophiala	0	1
**Total**	38	37

Supplementary Table S7 | Core fungal ASVs across a range of sample fractions. Computation is conducted across all samples (A) and the leaf surface (B). The leaf endosphere was omitted from this analysis since many LE samples failed. Core community composition is depicted in Figure 5.

A)

Fraction of samples [%]	ASV count	Fraction of community [%]
50	30	77.1
55	7	56.9
60	4	52.7

65	2	40.8
75	2	40.8
80	2	40.8
85	1	15.2

B)

Fraction of samples [%]	ASV count	Fraction of community [%]
50	128	92.1
55	112	90.6
60	102	87.3
65	88	86.7
70	80	86.1
80	59	82.5
85	45	80.8
90	38	79.0

95	29	76.9
100	2	3.5

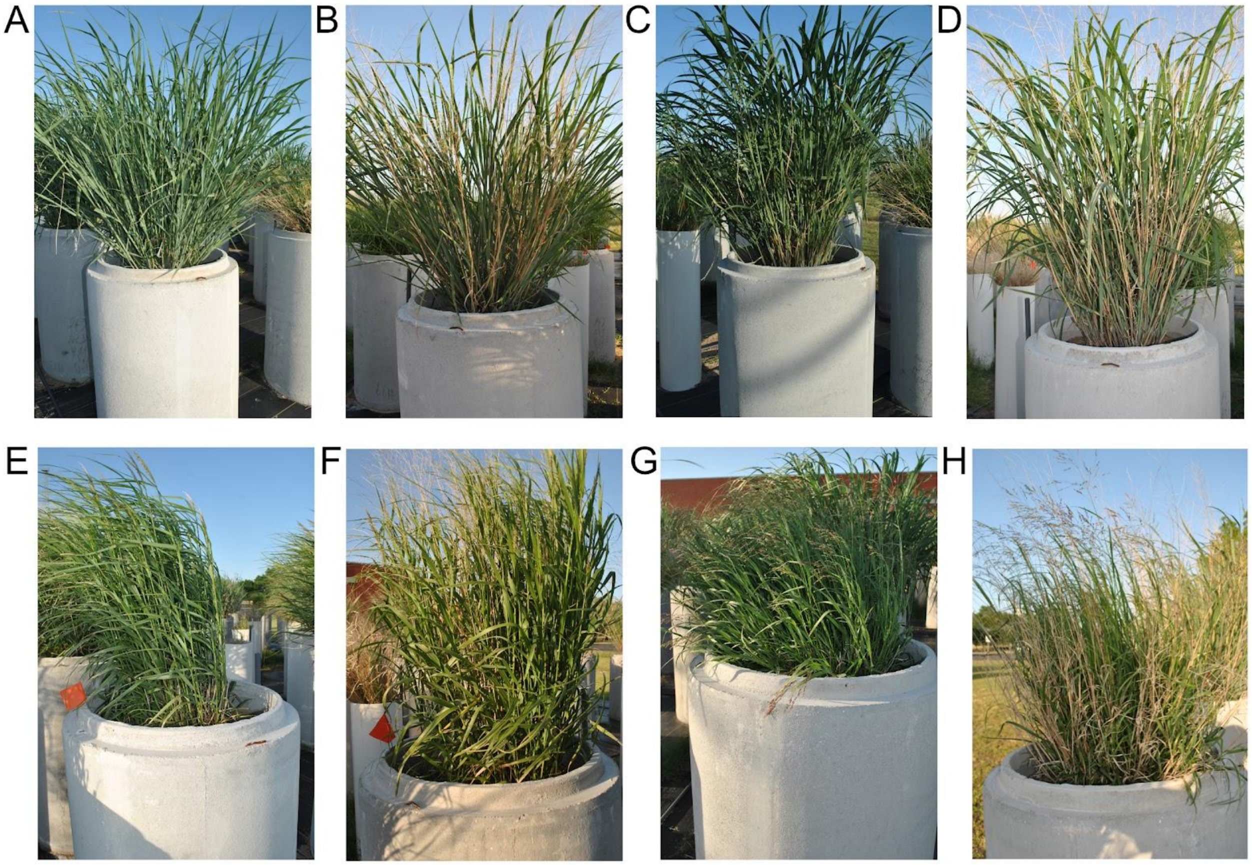

Figure S1 | Aboveground phenotypic differences between switchgrass plants from harvested and unharvested lowland (AP13, WBC) and upland (VS16, DAC) plants. (A) Harvested AP13, (B) unharvested AP13, (C) harvested WBC, (D) unharvested WBC, (E) harvested VS16, (F) unharvested VS16, (G) harvested DAC, (H) unharvested DAC.

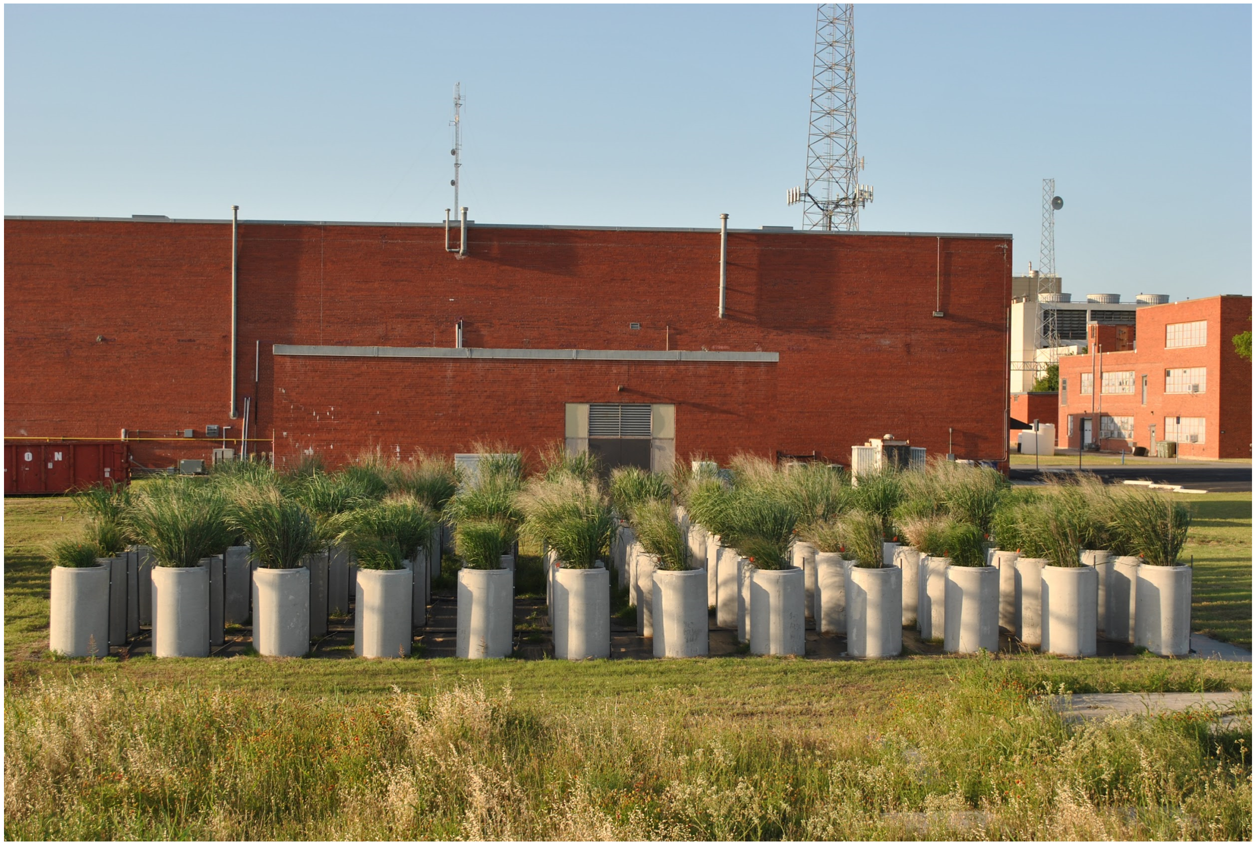

Figure S2 | Experimental setup. 96 plants were individually placed in concrete cylinders preventing exchange between plants.

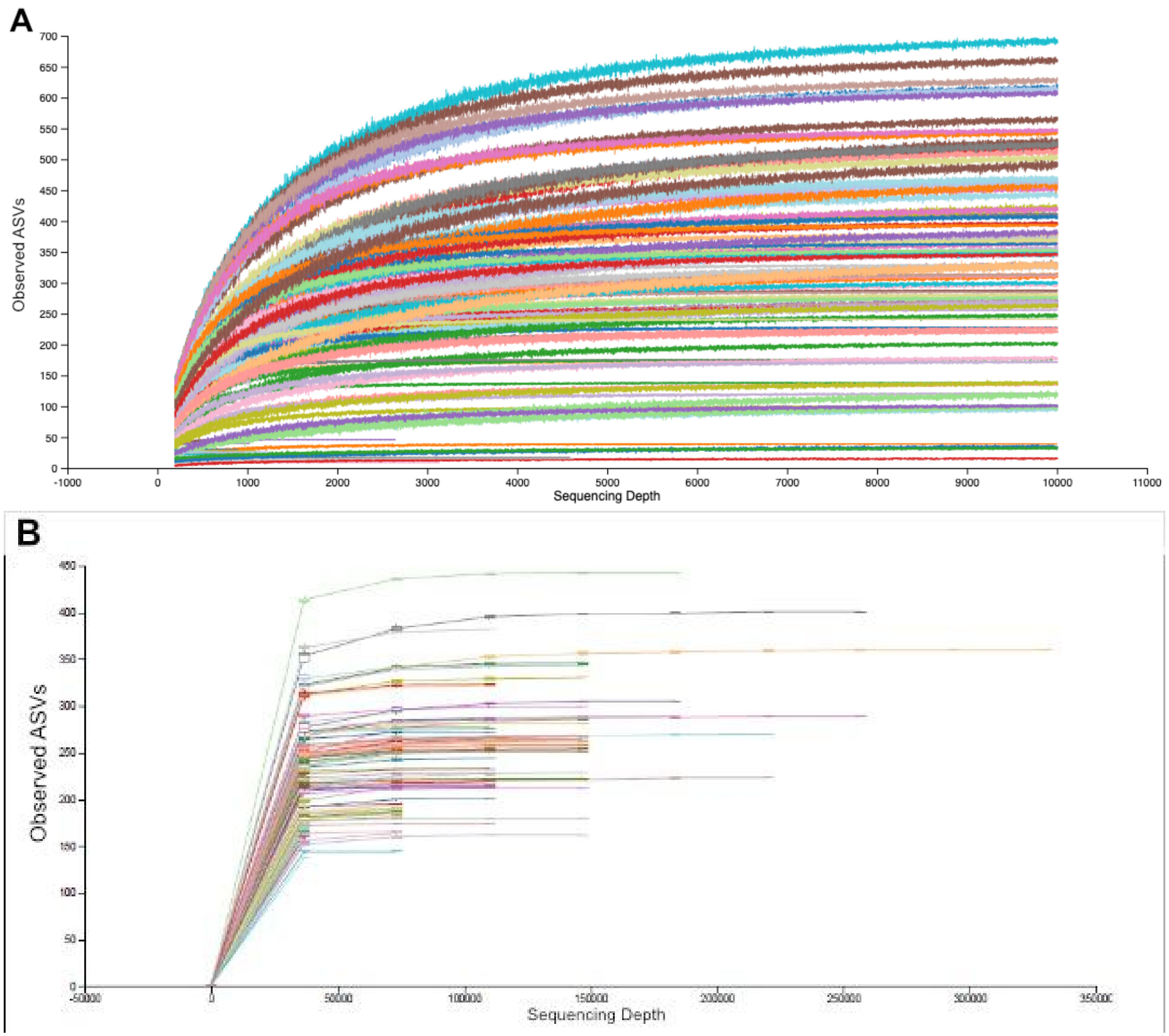

Figure S3 | Rarefaction curves based on A) 16S rRNA gene sequencing and on B) ITS sequencing.

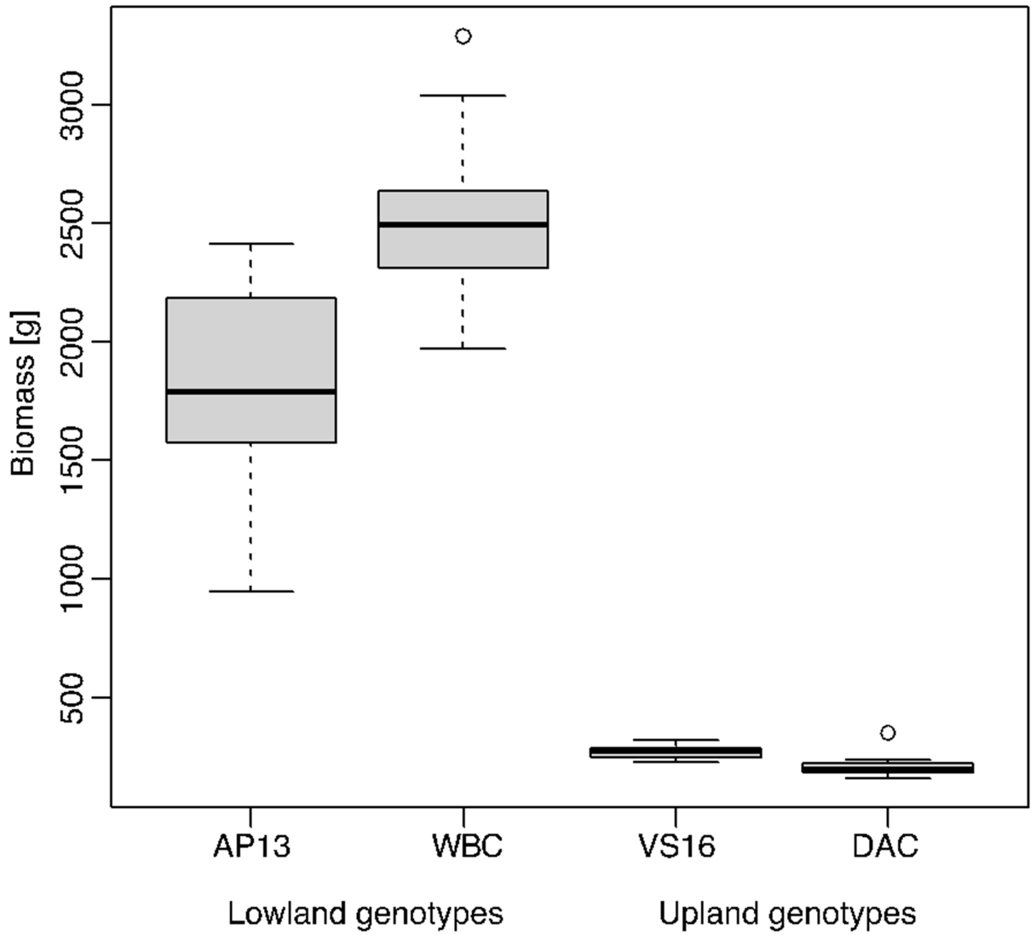

Figure S4 | Biomass differences between switchgrass genotypes/ecotypes.

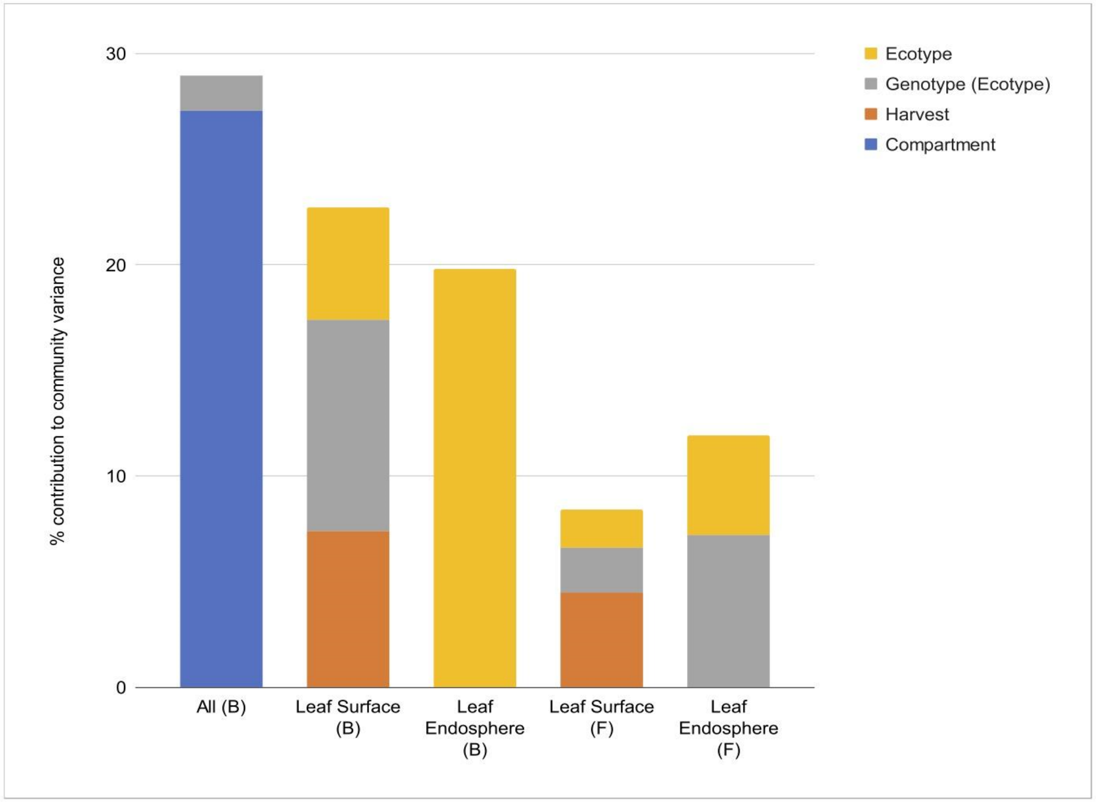

Figure S5 | Relative contribution of experimental factors to microbial community variance. (B) = Bacteria; (F) = Fungi.

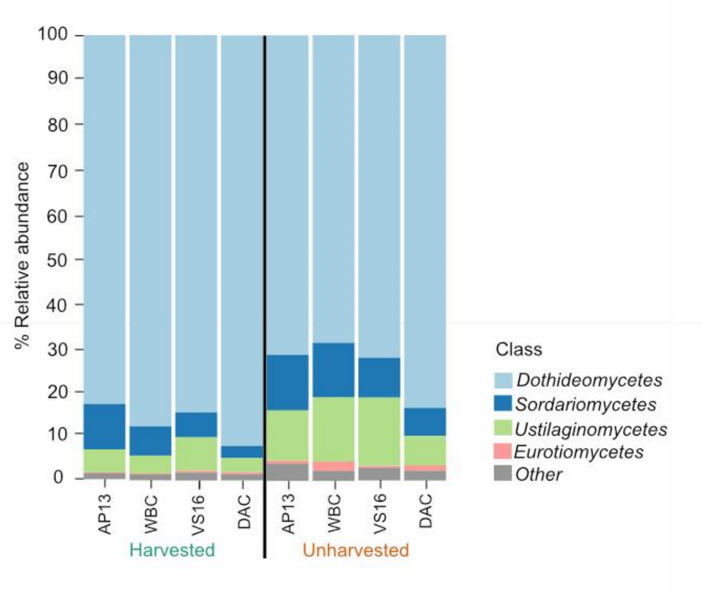

Figure S6 | Community composition of fungi in phyllosphere samples at the class level grouped by harvest level and genotype.

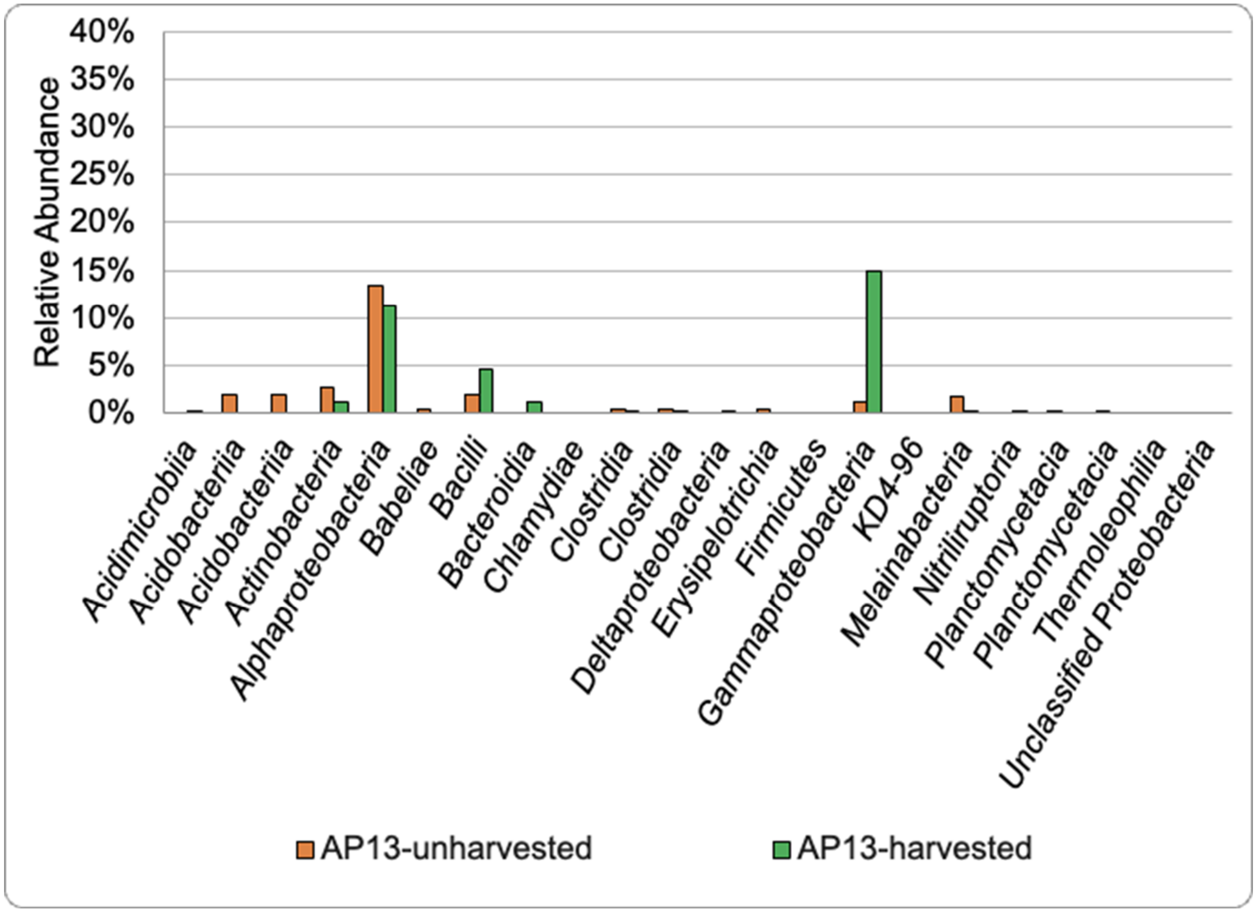

40%
35%
30%
25%
20%
15%
10%
5%
0%
Relative Abundance
Acidimicrobiia
Acidobacteriia
Acidobacteriia
Actinobacteria
Alphaproteobacteria
Babeliae
Bacilli
Bacteroidia
Chlamydiae
Clostridia
Clostridia
Deltaproteobacteria
Erysipelotrichia
Firmicutes
Gammaproteobacteria
KD4-96
Melainabacteria
Nitriliruptoria
Planctomycetacia
Planctomycetacia
Thermoleophilia
Unclassified Proteobacteria
AP13-unharvested
AP13-harvested

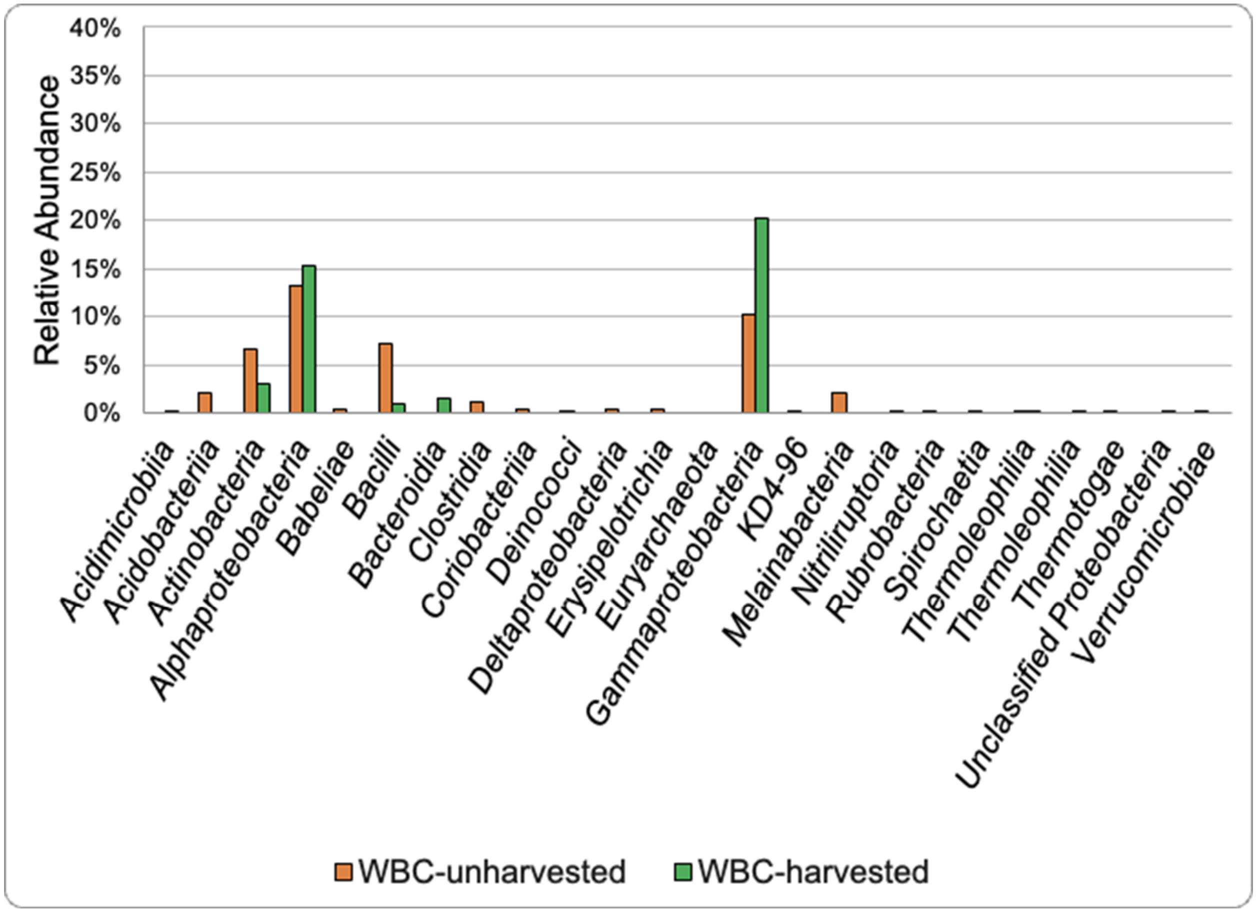

Relative Abundance
40%
35%
30%
25%
20%
15%
10%
5%
0%
Acidimicrobiia
Acidobacteriia
Actinobacteria
Alphaproteobacteria
Babeliae
Bacilli
Bacteroidia
Clostridia
Coriobacteriia
Deinococci
Deltaproteobacteria
Erysipelotrichia
Euryarchaeota
Gammaproteobacteria
KD4-96
Melainabacteria
Nitriliruptoria
Rubrobacteria
Spirochaetia
Thermoleophilia
Thermoleophilia
Thermotogae
Unclassified Proteobacteria
Verrucomicrobiae
WBC-unharvested
WBC-harvested

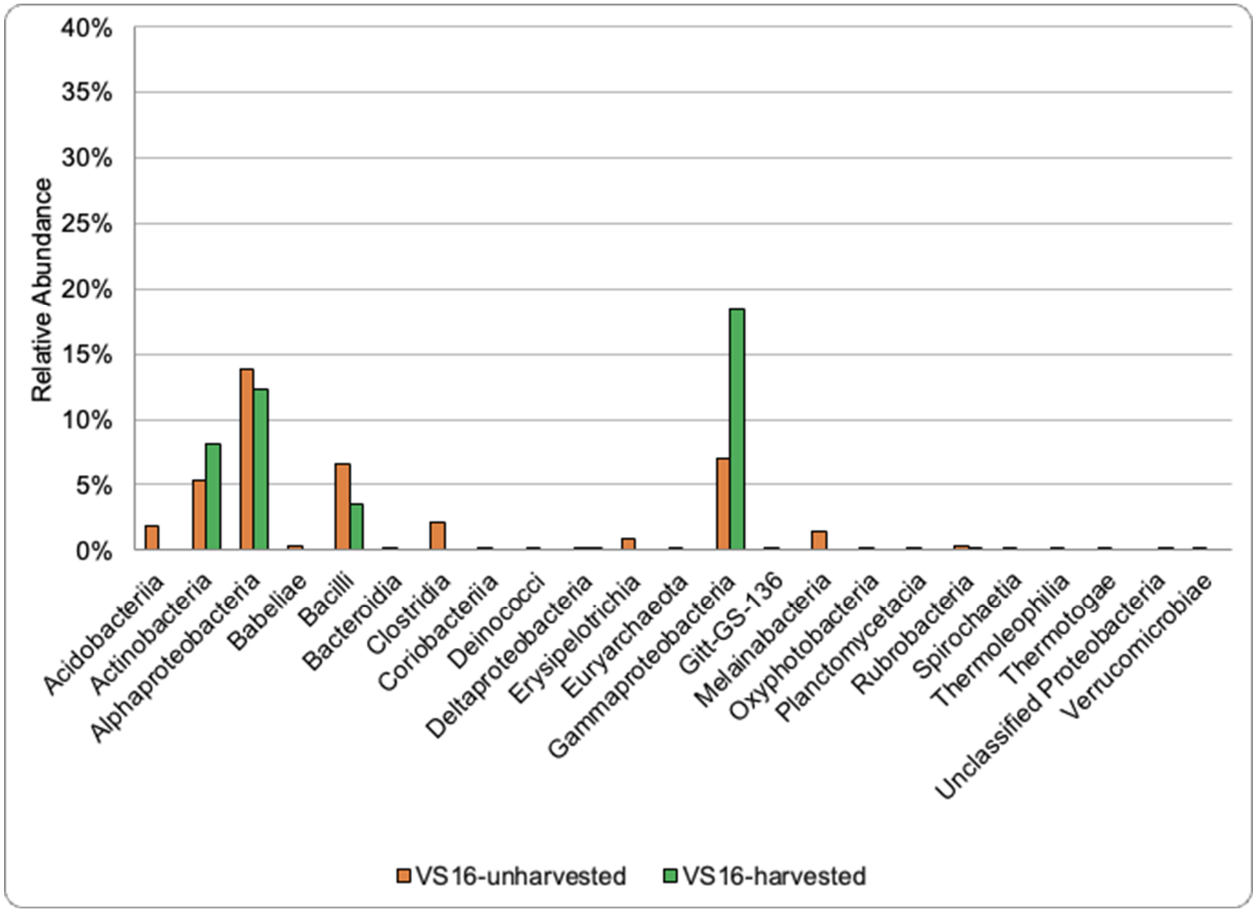
Relative Abundance
40%
35%
30%
25%
20%
15%
10%
5%
0%
Acidobacteriia
Actinobacteria
Alphaproteobacteria
Babeliae
Bacilli
Bacteroidia
Clostridia
Coriobacteriia
Deinococci
Deltaproteobacteria
Erysipelotrichia
Euryarchaeota
Gammaproteobacteria
Gitt-GS-136
Melainabacteria
Oxyphotobacteria
Planctomycetacia
Rubrobacteria
Spirochaetia
Thermoleophilia
Thermotogae
Unclassified Proteobacteria
Verrucomicrobiae
VS16-unharvested
VS16-harvested

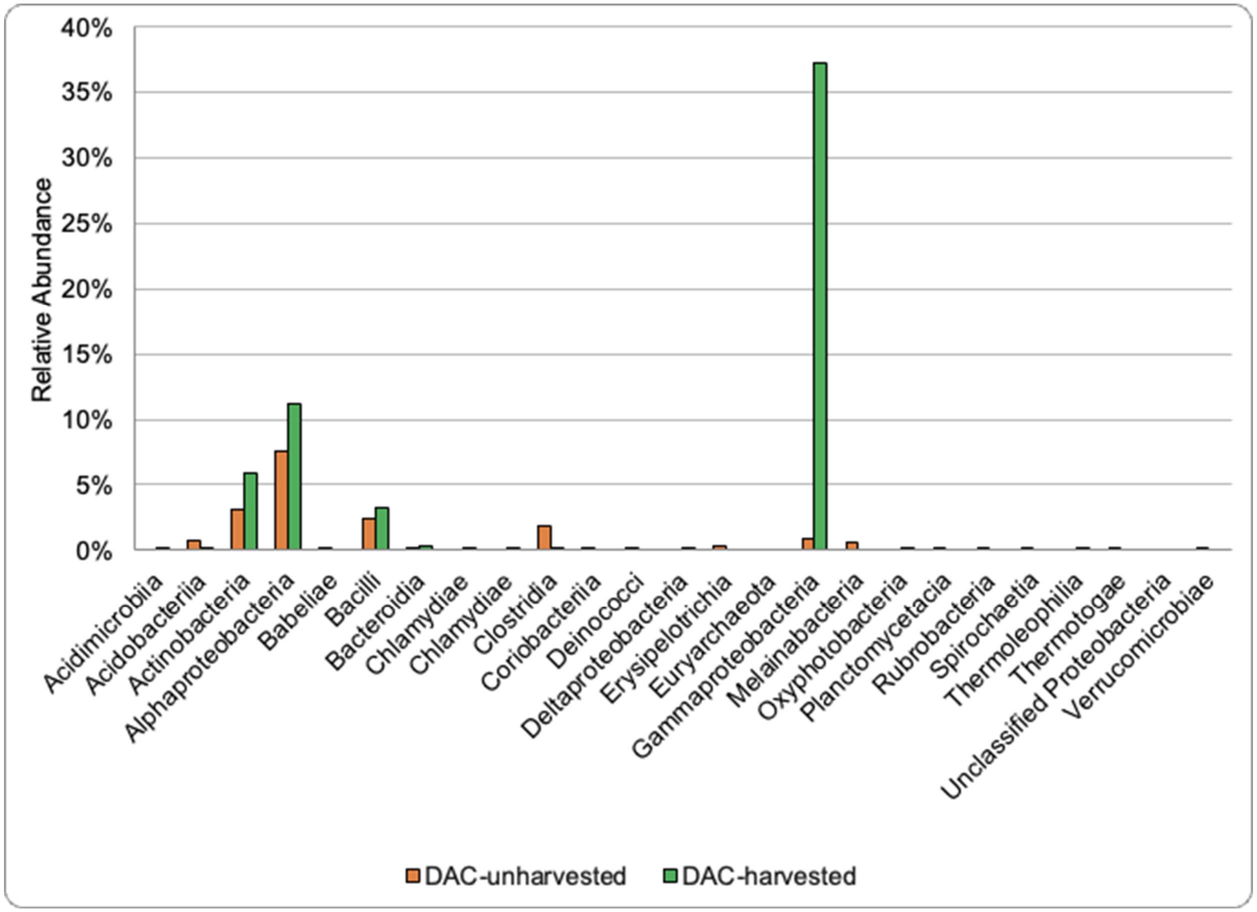

Figure S7 | Taxa enriched in harvested *vs*. unharvested switchgrass phyllosphere samples by genotype. A) AP13 genotype, lowland ecotype; B) WBC genotype, lowland ecotype; C) VS16 genotype, upland ecotype; D) DAC genotype, upland ecotype.

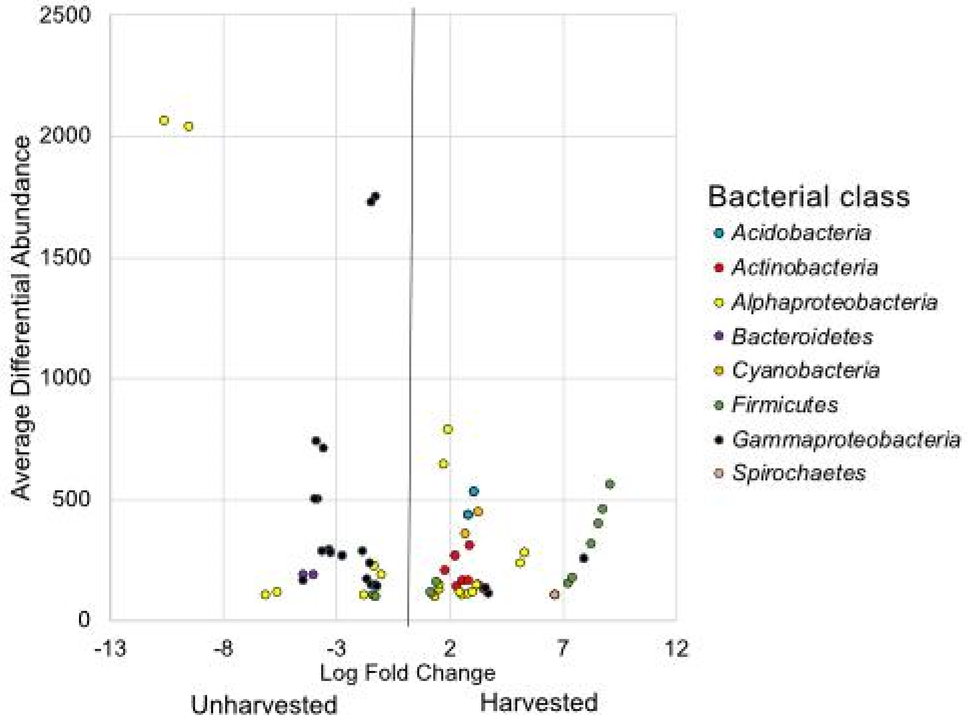

Figure S8 | Log fold change of significantly enriched bacterial ASVs by harvest level in the phyllosphere.

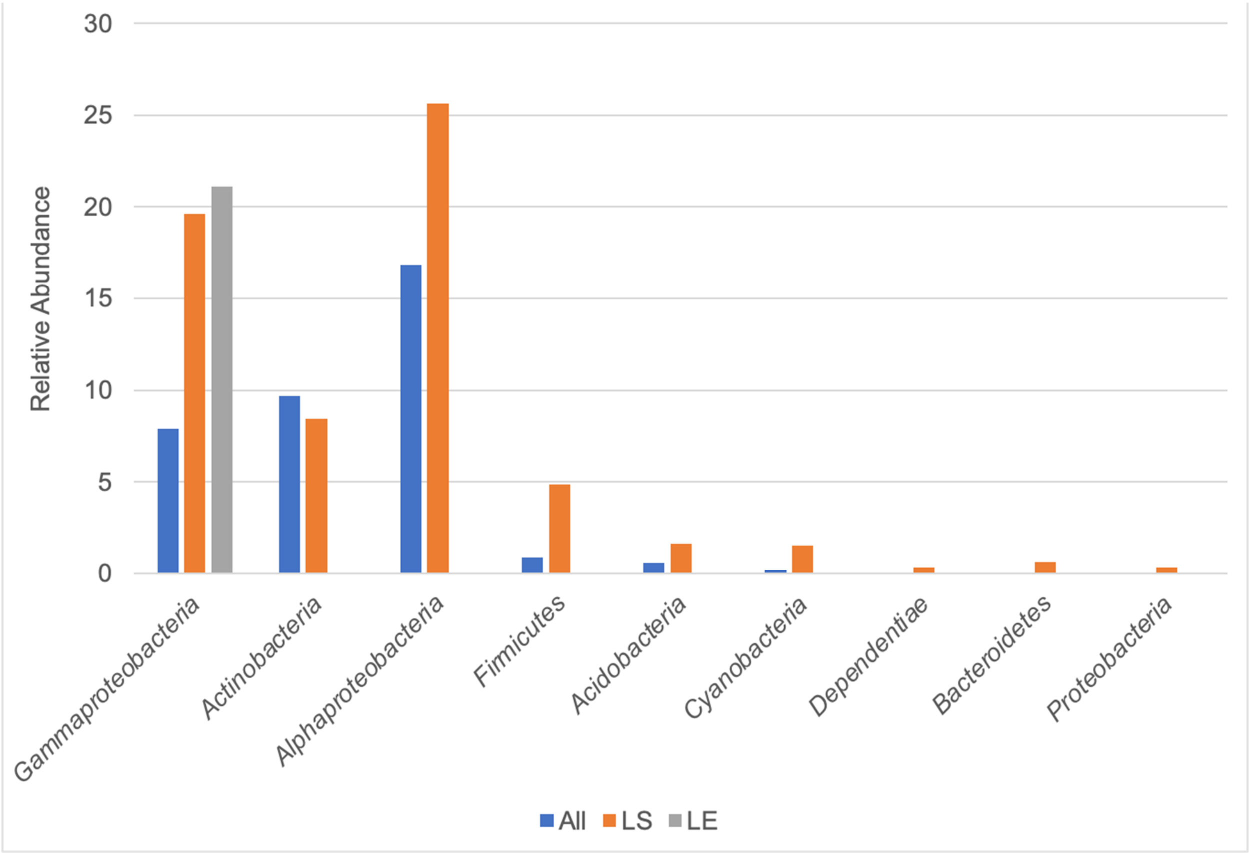

Figure S9 | Bacterial core microbiome composition shared among 50% of all, leaf surface (LS), and leaf endosphere (LE) samples.

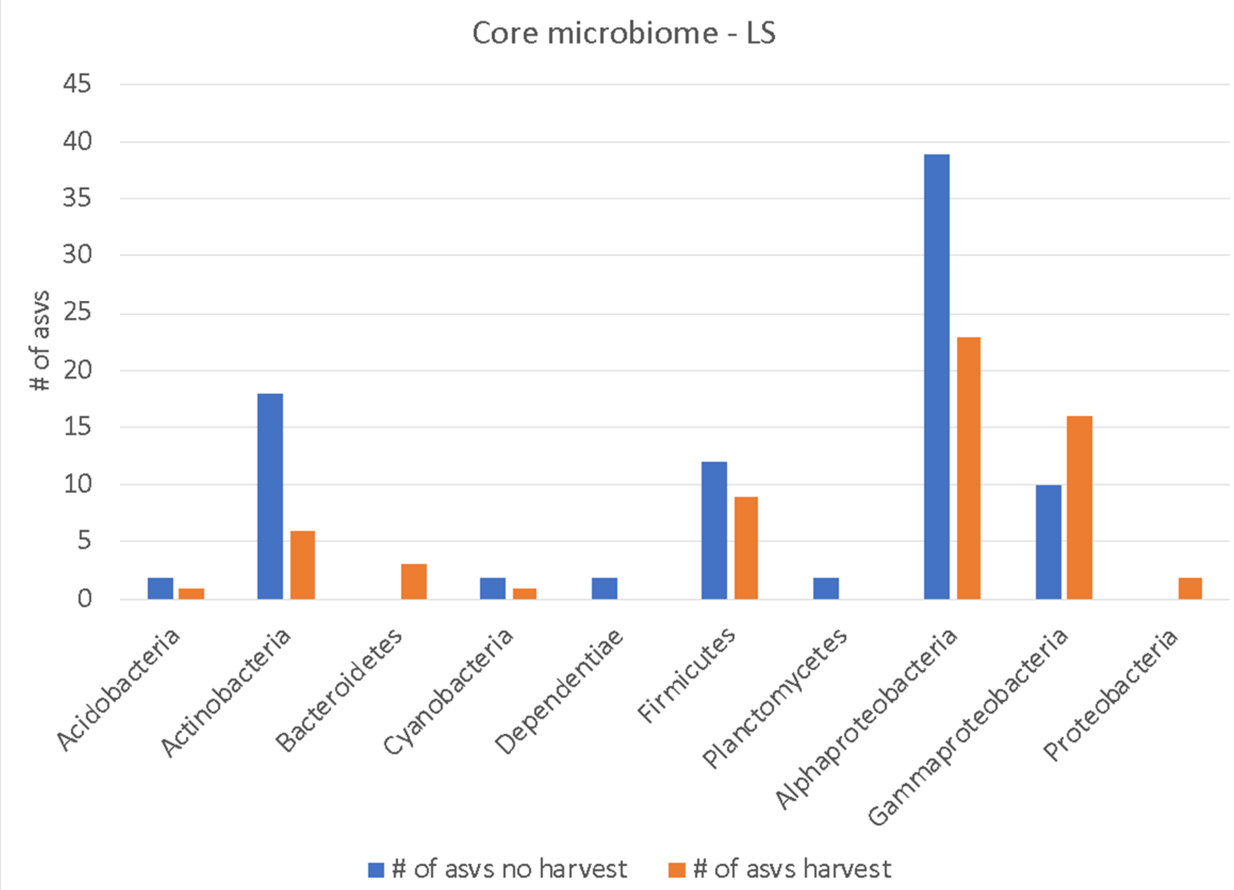

Figure S10 | Core microbiome in the phyllosphere by harvest level.

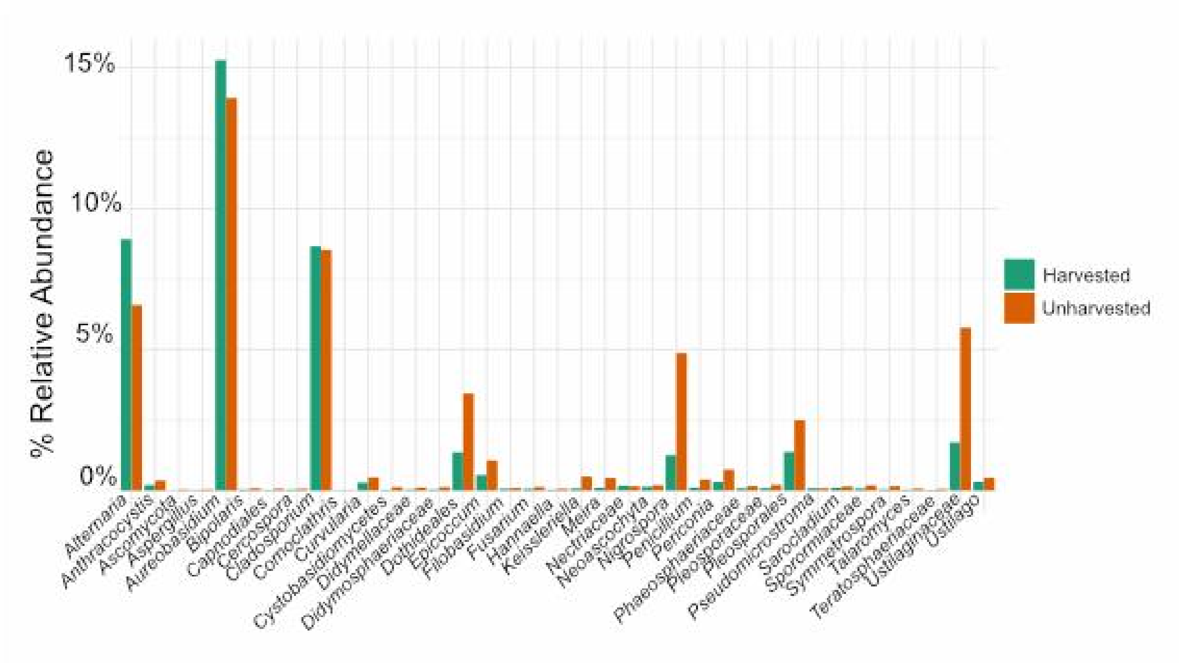

Figure S11 | Relative abundance of fungal taxa in harvested *vs.* unharvested phyllosphere samples.
